# Rectification of radiotherapy-induced cognitive impairments in aged mice by reconstituted Sca-1^+^ stem cells from young donors

**DOI:** 10.1186/s12974-019-1681-3

**Published:** 2020-02-07

**Authors:** Lukasz Wlodarek, Feng Cao, Faisal J. Alibhai, Adam Fekete, Nima Noyan, Stephanie W. Tobin, Tina B. Marvasti, Jun Wu, Shu-Hong Li, Richard D. Weisel, Lu-Yang Wang, Zhengping Jia, Ren-Ke Li

**Affiliations:** 1 Toronto General Hospital Research Institute, University Health Network, Toronto Medical Discovery Tower, Room 3-702, 101 College Street, Toronto, Ontario M5G 1L7 Canada; 2 Department of Physiology, Faculty of Medicine, University of Toronto, Toronto, ON Canada; 3 Program in Neurosciences & Mental Health, SickKids Research Institute, Floor 5, 555 University Avenue, Toronto, Ontario M5G 1X8 Canada; 4 Faculty of Medicine, Institute of Medical Science, University of Toronto, Toronto, ON Canada; 5 Department of Surgery, Division of Cardiac Surgery, University of Toronto, Toronto, ON Canada

**Keywords:** Radiotherapy, Aging, Microglia, Learning and memory, Bone marrow stem cells

## Abstract

**Background:**

Radiotherapy is widely used and effective for treating brain tumours, but inevitably impairs cognition as it arrests cellular processes important for learning and memory. This is particularly evident in the aged brain with limited regenerative capacity, where radiation produces irreparable neuronal damage and activation of neighbouring microglia. The latter is responsible for increased neuronal death and contributes to cognitive decline after treatment. To date, there are few effective means to prevent cognitive deficits after radiotherapy.

**Methods:**

Here we implanted hematopoietic stem cells (HSCs) from young or old (2- or 18-month-old, respectively) donor mice expressing green fluorescent protein (GFP) into old recipients and assessed cognitive abilities 3 months post-reconstitution.

**Results:**

Regardless of donor age, GFP^+^ cells homed to the brain of old recipients and expressed the macrophage/microglial marker, Iba1. However, only young cells attenuated deficits in novel object recognition and spatial memory and learning in old mice post-irradiation. Mechanistically, old recipients that received young HSCs, but not old, displayed significantly greater dendritic spine density and long-term potentiation (LTP) in CA1 neurons of the hippocampus. Lastly, we found that GFP^+^/Iba1^+^ cells from young and old donors were differentially polarized to an anti- and pro-inflammatory phenotype and produced neuroprotective factors and reactive nitrogen species in vivo, respectively.

**Conclusion:**

Our results suggest aged peripherally derived microglia-like cells may exacerbate cognitive impairments after radiotherapy, whereas young microglia-like cells are polarized to a reparative phenotype in the irradiated brain, particularly in neural circuits associated with rewards, learning, and memory. These findings present a proof-of-principle for effectively reinstating central cognitive function of irradiated brains with peripheral stem cells from young donor bone marrow.

**Electronic supplementary material:**

The online version of this article (10.1186/s12974-019-1681-3) contains supplementary material, which is available to authorized users.

## Introduction

Brain cancer remains one of the leading causes of death worldwide [1]. Current treatments using radiation are effective when applied with spatiotemporal control, but also lead to cognitive complications that arise later in life [2, 3]. Despite this, there is evidence to suggest that the preservation of cognitive abilities after radiotherapy is age-dependent [4]. Indeed, the brain’s regenerative capacity decreases with age and becomes more prone to injury over time [5]. Therefore, methods to reduce the development of impaired cognitive function in the aged population, especially after radiation therapy, are needed.

Although there are many reasons for cognitive capacity to decline with age, recent findings converge on microglial deterioration as being a key factor in this problem [6]. Microglia aging leads to a loss of neuroprotective properties and the acquisition of an inflammatory phenotype marked by increased secretions of pro-inflammatory chemokines and cytokines [7]. This “inflammaging” of microglia ultimately leads to exaggerated and detrimental responses following injury which impede proper functional recovery as a consequence of dysregulated inflammatory signalling [8]. In contrast, microglial cells in the young brain are not polarized to an inflammatory phenotype. Instead, they secrete neurotrophic factors which regulate neuronal function and repair processes [9, 10]. Collectively, this evidence suggests that rejuvenation of aged microglia to a younger phenotype may be a new approach to improve brain function in aged individuals.

Despite enormous advances in the field, the origin of microglia is still a debated topic to date. It is reported that the vast majority of microglia are derived from the embryonic yolk sac during development with little to no input from the periphery [11–13]. This hypothesis is further supported by several selective microglial depletion studies. Most notably, it was initially discovered that ablated microglia can repopulate from local nestin^+^ microglial progenitor cells [14]. Recently, this finding has been challenged as bone marrow (BM)-derived macrophages in the brain were also nestin^+^ [15]. Instead, this same group, and others, demonstrated that depleted microglia primarily repopulate from themselves rather than from local nestin^+^ microglial progenitor cells [15–17]. However, despite this, research evidence has also demonstrated that BM stem and progenitor cells can migrate to the brain under normal and pathological conditions, and subsequently give rise to microglial-like cells that express a combination of proteins similar to those expressed by brain resident microglia [18–20]. These BM-derived microglial-like cells have been observed to engraft in the brain under homeostatic conditions and maintain an identity distinct from resident microglial cells [21]. However, following forced depletion of microglia in the brain, a very recent study demonstrated that the repopulation of an empty microglial niche is the result of a combined effort and competition between local proliferating microglia and infiltrating monocyte-derived macrophages that have been classified as a functionally distinct subset of microglial-like cells [22]. Although the function of BM-derived Iba1^+^ cells remains unclear, these cells have recently been attributed towards tissue repair and restoration of function following injury [23, 24]. Interestingly, these beneficial effects are only observed when animals are recipients of young BM, but not old, presumably because old BM cells acquire a senescent-like phenotype upon migration to the injured brain [25]. Here, we hypothesize that the introduction of young microglia into the aged and irradiated brain may be beneficial for attenuating cognitive decline following radiotherapy.

In this study, we show that stem cell antigen-1 (Sca-1)-expressing cells repopulate the BM and give rise to cells which migrate to the aged and irradiated brain and differentiate into functional Iba1^+^ cells. Aged recipients of young Sca-1^+^ stem cells demonstrate significantly improved novel object recognition and spatial memory and learning following irradiation. In addition, we found that neurons in the hippocampus of aged mice who received young Sca-1^+^ stem cells possessed greater synaptic connectivity and plasticity. Taken together, our research evidence concludes that the introduction of Sca-1^+^ HSCs purified from young BM is a viable and promising approach in the amelioration of cognitive deficits that arise following radiotherapy of the aged brain.

## Methods

### Mice

Young (2-month-old) and middle-aged (12-month-old) C57BL/6-Tg(CAG-EGFP)1Osb/J and WT C57BL/6, as well as young NSG mice (NOD-*scid* IL2Rgamma^null^) were obtained from Jackson Laboratories. Mice were maintained and aged to 20–24 months within the animal housing facility at an ambient temperature of 21.5 °C. All mice were kept on a 12-h light/dark cycle and provided ad libitum access to both food and water. The Animal Care Committee of the University Health Network approved all experimental procedures, which were carried out according to the Guide for the Care and Use of Laboratory Animals (National Institutes of Health, 2011).

### Human samples

Male and female patients older than 18 years of age who were scheduled for non-emergency open heart surgery were consented for the study. Patients with known active malignancy within the past 3 years or simultaneous participation in another study with an investigational pharmacological agent were not recruited. Sternal BM was harvested the day of the surgery, under general anaesthesia. Patients’ chest skin were incised in the midline and an 18-gauge needle containing 5 mL of 10% heparin solution was advanced slowly through the periosteum of the sternum and rotated as it passed through the anterior table. The solution was injected into the sternum and the plunger was pulled back to aspirate between 15 and 40 mL of BM fluid from the sternum. The syringe containing the bone marrow was placed on ice and transported into the laboratory where mononuclear cells were separated by density gradient centrifugation using the Ficoll solution (Pharmacia, Piscataway, NJ). The isolated mononuclear cells were stained with PE-conjugated mouse anti-human CD34 (clone: 4H11, eBioscience) for cell isolation and reconstitution. The research ethics board of the UHN approved this investigation.

### Donor cell preparation

Both young and old (18–20-month-old) donor BM was obtained from the femurs and tibiae of male C57BL/6-Tg-GFP mice. The bones were flushed with PBS and then incubated in 5 ml erythrocyte lysis buffer (154.42 mM NH4Cl, 11.9 mM NaHCO3, 0.026 mM EDTA) for 5 min, followed by centrifugation at 1000 rpm for 5 min. The resulting pellet was suspended in Iscove’s modified Dulbecco’s medium (Thermo Fisher Scientific) and passed through a 40-μm filter. Cells were then counted and separated into Sca-1 positively labelled fractions using immunomagnetic activated cell sorting according to the manufacturer’s instructions (Stem Cell Technologies). For human cells, mononuclear cells were separated into positively and negatively labelled CD34 fractions using immunomagnetic activated cell sorting following the manufacturer’s instructions (STEMCELL Technologies), and the purity of positive cells was confirmed by flow cytometry. Functional capacity of CD34^+^ stem cells derived from human patients was evaluated using the colony-forming unit (CFU) assay for human HSCs (MethoCult H4034 Optimum, STEMCELL Technologies), followed by plating of 103 CD34^+^ cells in 35 mm dishes and cultured in the assay media. The number of CFU-GM, BFU-E, and total colonies was quantified 14 days post culture.

### BM reconstitution

Female C57BL/6 mice (18 months old) were lethally irradiated for 10 min at a rate of 1 Gy/min (10 Gy total, Cs-137 irradiator, Gammacell 40 Exactor) and then immediately given an injection (through the tail vein) with either young or old Sca-1^+^ cells (2 × 10^6^ cells), generating young Sca-1^+^ and old Sca-1^+^ chimera, respectively. Mice were then sacrificed 12 weeks later, and their brains were collected for different analyses (see methodology below). For generation of humanized mice, CD34^+^ cell fractions (0.7 × 10^6^ cells) from each patient were injected into irradiated 8–12-week-old female NSG mice via the tail vein to create human CD34^+^ reconstituted mice. The Animal Care Committee of the University Health Network approved all experimental procedures, which were carried out according to the Guide for the Care and Use of Laboratory Animals (NIH, 8th Edition, 2011). The mice were irradiated at 250, 285, and 325 cGy 24 h prior to injection.

### Mouse behavioural assays

The open-field test apparatus consisted of a 38 × 60 × 60 cm chamber with grey Plexiglas walls and transparent ceiling to allow for video recording. Mice (3 months post-reconstitution) were placed in the chamber for 10 min, and their ambulatory distance and rearing count was tracked and initially analyzed by idTracker [26], followed by additional analysis using a custom python script. The apparatus was cleaned with 70% ethanol between each mouse. The novel object recognition test was performed in the same chamber as the open-field test (see above) as previously described [27]. Briefly, mice underwent this test over the course of 3 days with each day separated by a whole 24 h. The first day (habituation phase) involved leaving mice to explore the chamber for 10 min. The chamber was cleaned with 70% ethanol between mice. The second day (familiarization phase) introduced two identical objects that were placed into adjacent corners of the chamber 5 cm away from the walls. The third day (testing phase) replaced one copy of the identical object with one copy of a novel object. Objects were randomized between mice to determine which would be the familiar set and which would be the novel set. Both objects were in the same spatial location as in day 2. For both the familiarization and testing phase, the mice were placed facing away from the objects, and the test was terminated once a total of 10 min was reached. The latter measurement was used to establish novel recognition memory by calculating object discrimination of total interaction time for the novel object over total interaction time for both novel and familiar objects. The Barnes maze consisted of an elevated circular platform (30 cm above the ground) with 20 equidistant holes around the circumference and was performed as described previously [28]. Briefly, a 10-cm barrier was constructed around the maze and two spatial cues were placed on its interior. One hole of the maze led to an escape chamber, which, when entered, resulted in the transportation of the animal to its home cage. Each animal ran the maze for a total of four consecutive days, four trials a day (20 min in-between trials), under a powerful light stimulus. The test was terminated when the animal entered the escape box or if 90 s of total exploration time was reached. If the termination condition was the latter, a clear beaker was placed over the animal and then the animal was gently guided towards the escape hole. Both primary completion time (the time taken to go near the escape hole and perform an extended head poke) and latency to escape box (the time taken when all four limbs entered the escape hole) were measured. Additionally, the number of errors that the animal made before finding the correct hole was also recorded.

### Immunostaining

Mice were anaesthetised with 2% isoflurane (Pharmaceutical Partners of Canada) prior to receiving a cardiac perfusion with PBS. Whole brains were extracted and fixed in 2% paraformaldehyde overnight at 4 °C before cryoprotection in sequential changes in 10%, 20%, and 30% (w/v) sucrose in PBS for 24 h each. Brains were embedded in O.C.T. (Tissue-Tek, Sakura), sectioned coronally at a thickness of 20 μm on a freezing-sliding microtome, and then stored at − 20 °C. Frozen tissue sections were blocked with 5% (w/v) donkey serum before incubation at room temperature for 2 h with primary antibodies at the following concentrations: NeuN, 1:100, MAB377, Millipore; GFAP, 1:200, Z0334, Dako; CD140a, 1:200, 558,774, BD Pharmingen; Iba1, 1:400, PA5-27436, Thermo Fisher Scientific; GFP, 1:400, A21311, Life Technologies; TH, 1:400, Ab112, Abcam; iNOS, 1:200, N32030/L19, BD Transduction Laboratories; Arg-1, 1:200, SC-18351, Santa Cruz Biotech; Ki-67, 1:50, Ab833, Abcam; Caspase-3, 1:200, 9665S, Cell Signalling; IGF-1, 1:200, ab9572, Abcam; FGF2, 1:200, SC-1360, Santa Cruz; GluR1, 1:200, ab31232, Abcam. Incubation with respective Alexa 488, 568, or 647 conjugated secondary antibodies was carried out at room temperature for 1 h with light protection. Cell nuclei were identified with 4′,6-diamidno-2-phenylindole (DAPI) at a 1:2000 dilution for 5 min. Cell quantification carried out on specific structures included all of the positive cells in that particular structure of interest. Randomly selected regions of cell quantification (1.0-mm^2^ cross sections) were only carried out when a total cell count was to be determined independent of any particular structure or region. Fluorescent images were obtained with a Nikon Eclipse Ti fluorescent microscope using a VS120 slide scanner. Mean fluorescence intensity was carried out using ImageJ (image processing and analysis software in Java) by calculating mean grey values of fluorescent signal for each separate channel across identically sized cross-sectional areas of brain tissue. Prior to analysis, images were captured at an identical exposure and set to and fixed fluorescence intensity using the Olympus cellSens imaging software. Proportion and number of cells were quantified automatically using built-in cell counting tools in the Olympus cellSens imaging software.

### RNA extraction and RTqPCR

Total RNA was isolated from mouse whole-brains using Tri-Reagent (Sigma-Aldrich) according to the manufacturer’s instructions. Reverse transcriptase was performed using SuperScriptIII (Invitrogen) and 1 μg of total RNA served as a template for each reaction. cDNA expression was analyzed using SensiFast (Bioline) SYBR Green with the following parameters: 95 °C for 2 min (95 °C for 5 s; 60 °C for 30 s for 40 cycles). Relative expression levels were normalized to glyceraldehyde 3-phosphate dehydrogenase (GAPDH).

### Protein extraction and western blotting

Total protein was extracted from homogenized whole brains in lysis buffer. For western blotting, 50 μg of lysate was fractioned through a 4% stacking and 10% running SDS-PAGE followed by transfer to a polyvinylidenediflouride membrane. Membranes were blocked with 5% (w/v) milk for 1 h at room temperature followed by incubation in the primary antibodies at 4 °C overnight at the following concentrations: TH, 1:2000, Ab112, Abcam; GluR1, 1:5000, ab31232, Abcam. PSD-95, 1:1000, D27E11, Cell Signaling; GAPDH, 1:5000, MAB374, Millipore. Membranes were then incubated with horseradish peroxidase-conjugated secondary antibody (SC-2030, 1:2000, Santa Cruz) for 1 h at room temperature. Visualization was performed with enhanced chemiluminescence. Images were processed and analyzed by ImageJ (image processing and analysis software in Java). The relative intensity of the bands was normalized to GAPDH.

### Enzyme-linked immunosorbent assay (ELISA)

Total protein was extracted from the hippocampus, cortex, and cerebellum, as described above. Soluble factor detection and quantification was carried out according to the manufacturer’s instructions (IFG1 Mouse ELISA Kit, ab100695, Abcam; FGF basic Mouse ELISA kit, ab100670, Abcam).

### In situ end labelling of DNA fragmentation (TUNEL)

For TUNEL staining, brains were removed as before and sectioned coronally at a thickness of 20 μm. Four serial sections of the hindbrain were processed per animal. Sections were then incubated for 2 h at 37 °C using the In Situ Cell Death Detection Kit, TMR Red (Sigma-Aldrich), according to the manufacturer’s instructions. Sections were then incubated for 5 min in 1:2000 DAPI followed by application of mounting media and coverslip. Viewing of DNA fragmentation was performed with a Nikon Eclipse Ti fluorescent microscope under the TRITC filter.

### Dendritic spine density analysis

To study the quantity and morphology of dendritic spines of pyramidal cells in the CA1 region of the hippocampus, brains were removed from the animals and incubated in a series of solutions using the FD Rapid GolgiStain kit (FD Neurotechnologies), according to the manufacturer’s instructions. Following cryoprotection of the whole brains with Solution C (see kit), tissue was sectioned coronally at 200 μm thickness and then viewed under a light microscope. Dendritic arbors of each cell were analyzed as previously described [29]. The spine density was calculated as the number of spines (defined as protrusions of the dendritic membrane regardless of shape) along a 30-μm dendritic terminal segment. A total of 10 different neurons were analyzed for each animal per group.

### Electrophysiology

All electrophysiological recordings were performed at the Schaffer/Collateral pathway in the hippocampus. The mouse brains were quickly removed to ice-cold artificial cerebrospinal fluid (ACSF) saturated with 95% O_2_/5% CO_2_, and then cut into sagittal 350 μm hippocampal slices. The ice-cold ACSF contained (in mM): 124 NaCl, 3 KCl, 26 NaHCO_3_, 1.25 NaH_2_PO_4_, 2 MgSO_4_, 10 d-glucose, and 2 CaCl_2_. Slices were recovered in 95% O_2_/5%CO_2_ saturated ACSF at 32–34 °C for 30 min and then maintained at room temperature for at least 1 h before recording. A single slice was then transferred to a recording chamber and superfused with 95% O_2_/5% CO_2_ saturated ACSF. Perfusion flow rate was at 2 ml/min. Synaptic transmission was recorded with glass pipettes (3–4 M Ω) filled with ACSF and evoked by stimulation at 0.05 Hz of the Schaffer/Collateral pathway in the hippocampus. For input/output experiments, the stimulus intensities range from 5, 10, 20, 40, 60, 80, 100, 120, 140, to 150 mV. For paired pulse field recordings, the inter-stimulus intervals range from 25, 50, 100, 200, 300, 400, 500, to 1000 ms. For comparison of the magnitude of long-term potentiation (LTP) between different groups, the mean values of the last 10 min of recordings were calculated and compared statistically. We recorded action potentials from GFP^+^ Purkinje cells in current-clamp configuration using K^+^-based intracellular solution containing (in mM): 97.5 K-gluconate, 32.5 KCl, 40 HEPES, 1 MgCl2, 0.5 EGTA, and pH = 7.2 was set by KOH. All data acquisition and analysis were done using pClamp 10 software (Molecular Devices, USA). The representative traces were averages of five successive sweeps during recording. *n* represents the number of hippocampal slices used in each experiment, with at most two slices per mouse used for each experiment.

### Microarray and bioinformatics

Total RNA was extracted from whole-brain mouse tissue using Tri-Reagent after Sca-1^+^ or Sca-1^−^ reconstitution as described above (*n* = 3). Quality control and microarray analysis was performed by Princess Margaret Genomics Centre (Toronto, Canada). Briefly, RNA quality was assessed by Agilent 2100 BioAnalyzer (RIN > 7.5). Gene expression was assessed using the Agilent SurePrint G3 8x60K mouse microarray. GeneSpring GX was used for data normalization and filtering using default parameters. Differentially expressed genes were identified using an unpaired *t*-test (*P* ≤ 0.05). A heat map of differentially expressed genes (fold change ≥ ± 1.5) was generated by RStudio. A volcano plot was generated by GeneSpring. Gene Ontology (GO) analysis was completed using the Database for Annotation, Visualization, and Integrated Discovery (DAVID).

### Statistics

All values are expressed as mean ± s.e.m. Analyses were performed using GraphPad Prism 6.0 software. Unpaired *t*-test was used for two-group comparison. Statistical significance of RTqPCR data, cell quantification, and behavioural data was carried out using an unpaired, two-tailed *t*-test. Three-group comparison was carried out using one-way analysis of variance (ANOVA) followed by Tukey post hoc tests and two-way ANOVA for two-factor variables followed by Tukey post hoc tests. Differences were considered statistically significant at *P ≤ 0.05; **P ≤ 0.01; ***P ≤ 0.001; †††† or ****P ≤ 0.0001.

## Results

### BM cells which arise from Sca-1^+^ stem cells migrate to the brain and give rise to Iba1^+^ cells

**Fig. 1:**
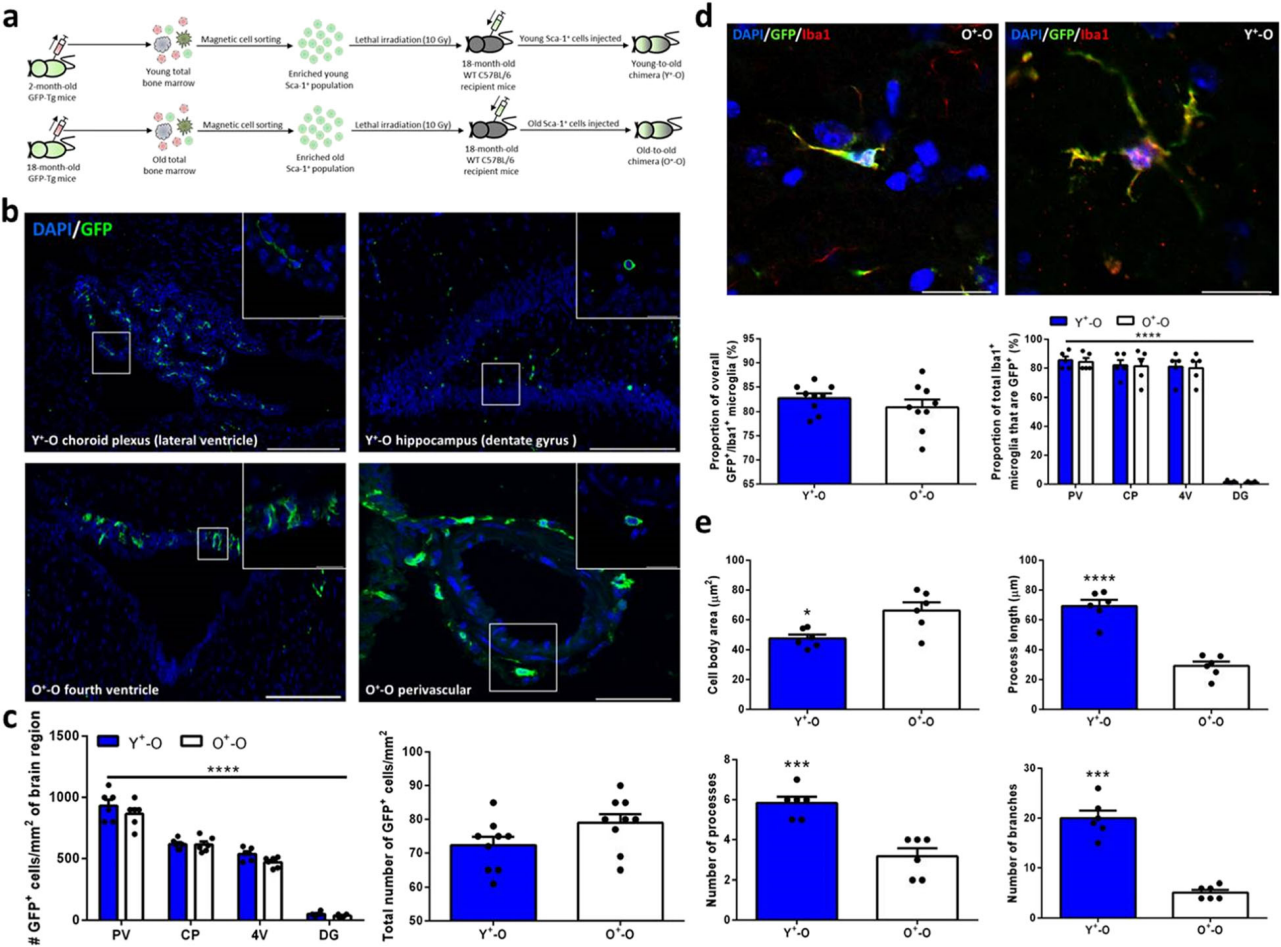
Reconstituted Sca-1^+^ stem cells home from the BM to the brain and differentiate into microglia. **a** Cartoon representation of the BM reconstitution process and chimera generation. **b** Immunostaining and **c** quantification of GFP^+^ cells in different brain regions with at least *n* = 6 mice per group. **d** Co-labelling and quantification of GFP^+^ cells with the microglial marker, Iba1, for at least *n* = 5 mice per group. **e** Quantification of GFP^+^/Iba1^+^ cell morphology for *n* = 5 mice per group. Scale bars, 200 μm and 20 μm for insets (**b**) and 20 μm and 40 μm for left and right images, respectively (**c**). Data are mean ± s.e.m. *P ≤ 0.05; ***P ≤ 0.001; ****P ≤ 0.0001 (unpaired two-sided t-tests (**c** (right), **d** (left), **e**), two-way ANOVA with Tukey’s multiple comparisons test (**c** (left; F (3, 31) = 339.9, *P* < 0.0001), **d** (right; F (3, 32) = 284.0, *P* < 0.0001)))

Stem cell therapies using cell transplantation for the repair and regeneration of brain tissue have been previously demonstrated to elicit beneficial effects; however, the positive effects are short-lived due to rapid loss of transplanted cells [30]. To establish a potentially long-term and continuous supply of cells to the brain, we reconstituted the BM of old WT C57BL/6 mice with an enriched population of either young or old GFP^+^ HSCs expressing Sca-1, generating young-to-old (Y^+^-O) and old-to-old (O^+^-O) chimeras, respectively (Fig. 1a). Three months post-reconstitution, GFP^+^ cells were seen in the brains of both groups, with the greatest density of these cells present in regions of highly permeable microvasculature (i.e., circumventricular organs and choroid plexus), perivascular tissue, and to a lesser extent the hippocampus (Fig. 1b, c). A large number of GFP^+^ cells were also present in the cerebellum, and occasionally, there were a small number of functional GFP^+^ Purkinje cells capable of eliciting normal action potentials (Additional file 1: Figure S1a, b). Aside from this, nearly all of the GFP^+^ cells in both groups were positive for the microglial marker, Iba1 (Fig. 1d). Morphologically, GFP^+^/Iba1^+^ cells derived from young donors were significantly larger in size with many more processes and branches relative to those originating from old donors (Fig. 1e).

### Young Sca-1^+^ stem cells recover radiation-induced deficits in learning and memory

Microglia have been previously implicated in learning and memory [31], synaptic plasticity [32], and neurogenesis [33] and possess an increased tendency to acquire inflammatory phenotypes with age [34, 35]. In order to investigate the effects of age on behaviour and microglial function in the absence of radiation, we examined memory and learning, as well as microglial morphology and activated state in young and old WT mice. In line with past findings [36, 37], we found overall more Iba1^+^ cells and a greater proportion of pro-inflammatory activated microglia in the brains of aged mice (Additional file 2: Figure S2a, b). In addition, we were able to detect performance differences between young and old mice on spatial memory maze completion times, but did not detect compromised object recognition in aged mice relative to their younger counterparts (Additional file 2: Figure S2c).

**Fig. 2:**
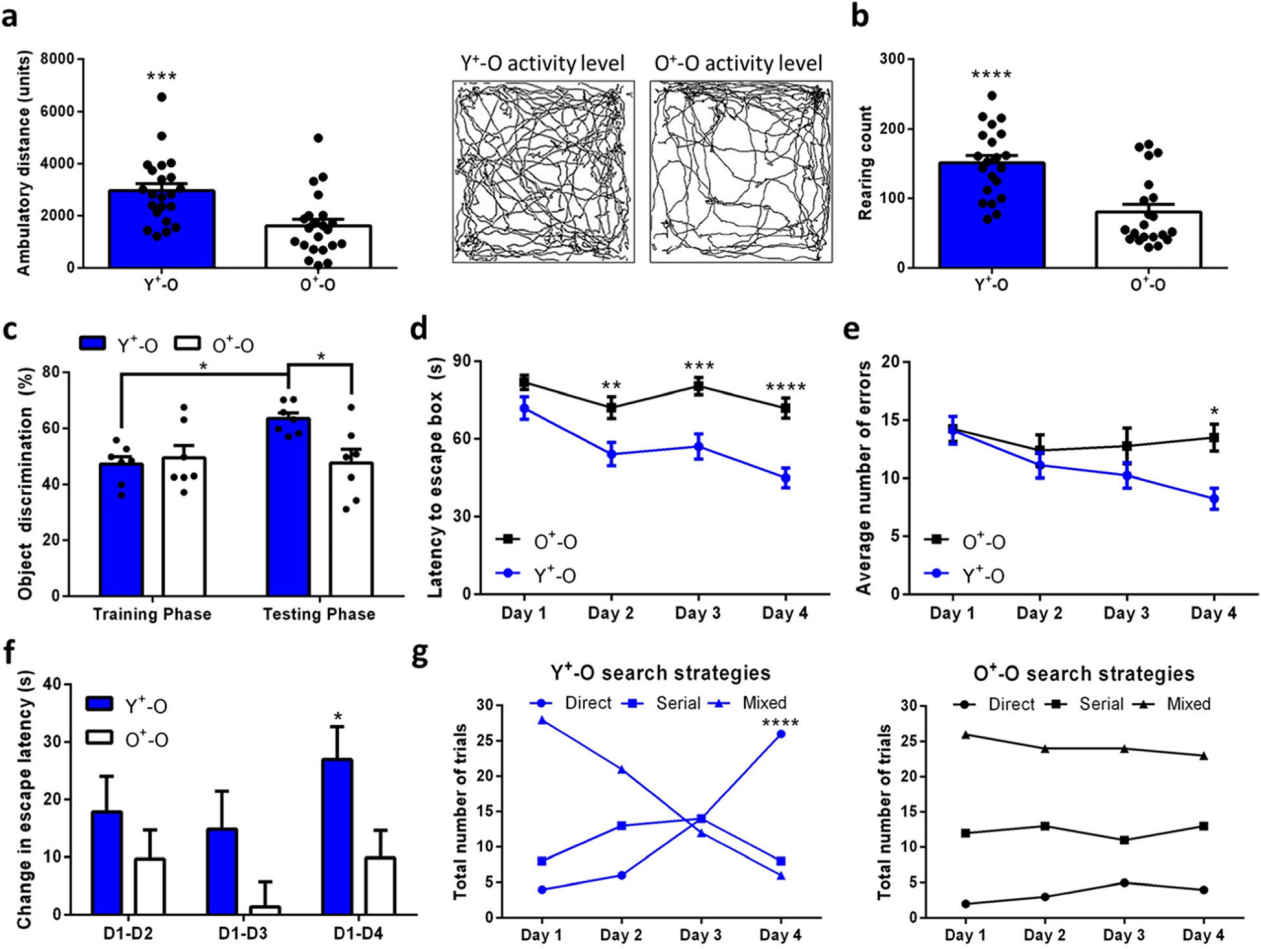
Old mice reconstituted with young Sca-1^+^ stem cells show improved novel object recognition and spatial memory and learning. **a** Activity level and **b** rearing count were recorded for 10 min in a novel environment with *n* = 22 mice per group. **c** Novel object recognition for *n* = 7 mice per group. Training and testing phases were separated by 24 h. **d** Barnes maze completion time and **e** frequency of mistakes for *n* = 10 mice per group. **f** Spatial learning between test days and **g** problem solving strategies for *n* = 10 mice per group. Data are mean ± s.e.m. *P ≤ 0.05; **P ≤ 0.01; ***P ≤ 0.001; ****P ≤ 0.0001 (unpaired two-sided *t*-tests (**a**, **b**), two-way ANOVA with Tukey’s multiple comparisons test (**c** (F (1, 24) = 6.103, *P* < 0.0201), **d** (F (1, 292) = 45.89, *P* < 0.0001), **e** (F (1, 41) = 4.940, *P* = 0.0293), **f** (F (1, 222) = 7.953, *P* = 0.0052) and chi-square test (**g**))

Since cognitive impairments have been previously described to manifest earlier and with greater severity in geriatric patients following radiotherapy, as opposed to paediatric patients [38, 39], we investigated whether our newly introduced young BM-derived Iba1^+^ cells could benefit aged and irradiated animals on a behavioural level. Using the exploratory initiative/curiosity paradigms, we found that Y^+^-O mice displayed greater ambulatory distance and rearing count when compared to O^+^-O mice (Fig. 2a, b). Since rearing behaviour is influenced by the hippocampus [40], we next investigated hippocampal-dependent spatial reference and novel object recognition memory using the Barnes maze and novel object recognition task, respectively. Relative to old mice given aged Sca-1^+^ stem cells, we found that mice given young Sca-1^+^ stem cells could learn to distinguish familiar objects from novel objects (Fig. 2c), as well as demonstrated improved spatial learning and error correction by using different learning strategies during maze performance for up 3 months after irradiation (Fig. 2d–g).

### Young Sca-1^+^ stem cells activate genes involved in memory and neurodevelopment

**Fig. 3:**
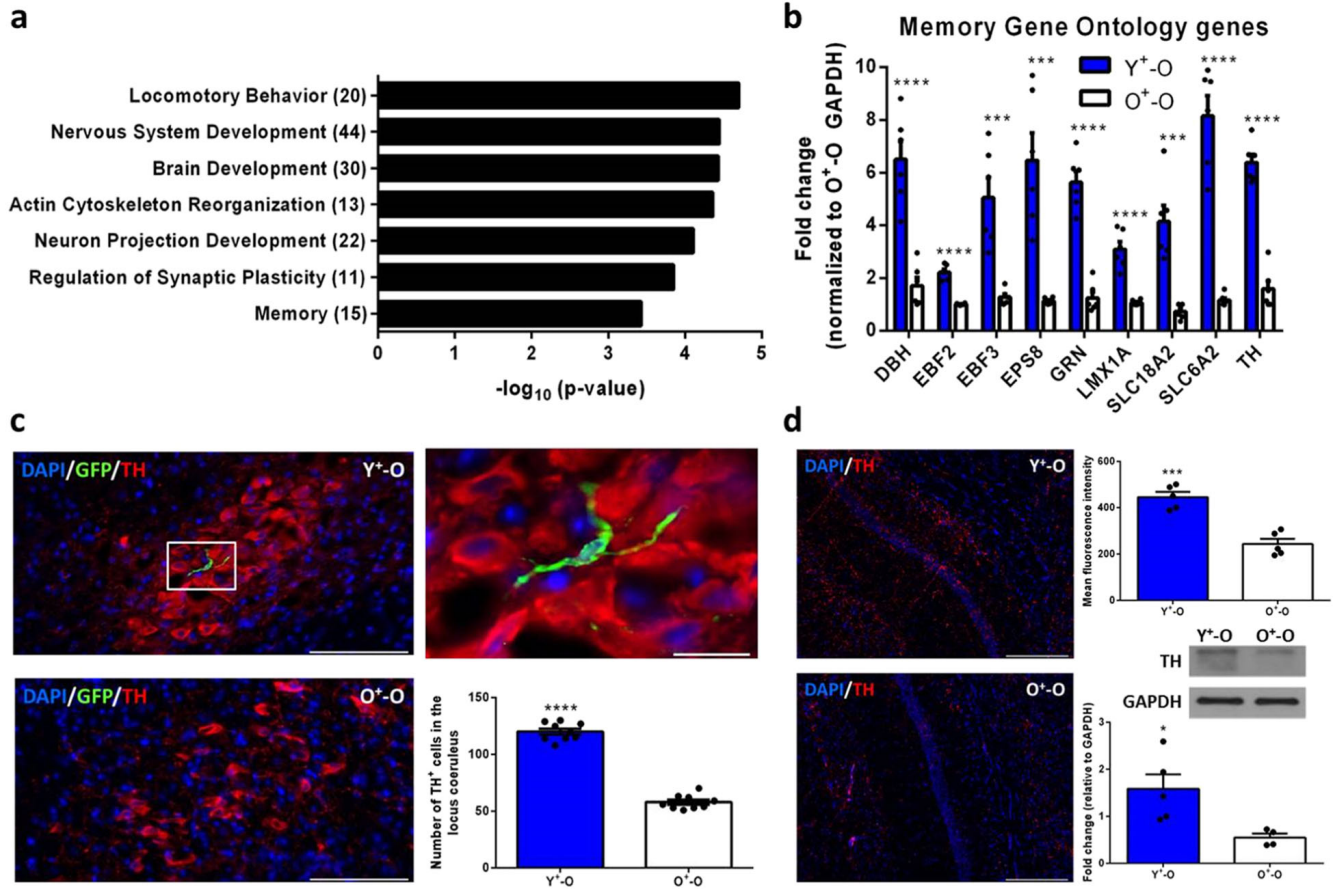
Young Sca-1^+^ stem cells upregulate several dopamine-dependent memory genes and preserve neuronal numbers in the locus coeruleus. **a** Significantly dysregulated genes organized according to function and significance using Gene Ontology (Y^+^-O vs. Y^−^-O) for *n* = 3 animals per group. **b** Verification of several significantly upregulated memory genes using RTqPCR for *n* = 5 animals per group. **c** Immunostaining for dopaminergic neurons expressing tyrosine hydroxylase in the locus coeruleus and **d** tyrosine hydroxylase^+^ tracts in the CA1 hippocampus with representative western blot analysis on the whole hippocampus for at least *n* = 5 mice per group. Scale bars, 200 μm (**c**, **d**) and 20 μm for inset (**c**). Data are mean ± s.e.m. *P ≤ 0.05; ***P ≤ 0.001;****P ≤ 0.0001 (unpaired two-sided *t*-tests (**b**, **c**, **d**))

To better understand how the introduction of peripherally derived stem cells into the brain affects overall learning and memory, we carried out a genome-wide microarray analysis on aged whole brains of mice that either received young Sca-1^+^ stem cells or young BM cells devoid of Sca-1^+^ stem cells (Sca-1^−^) 3 months post-irradiation. We observed extensive differential gene expression between the two groups (Additional file 3: Figure S3a, b) and found that young Sca-1^+^ stem cells (relative to young Sca-1^−^ cells) significantly upregulated many neurodevelopmental and memory genes relating to dopaminergic and adrenergic systems (Additional file 3: Figure S3c; Figure S3a), as verified by RTqPCR (Fig. 3b). From the brain areas searched relating to these two systems, we found a large number of GFP^+^ cells surrounding the locus coeruleus, supposedly due to its close proximity with the blood-brain barrier-devoid fourth ventricle, with a significantly greater number of tyrosine hydroxylase-expressing (TH^+^) dopaminergic neurons in Y^+^-O mice (Fig. 3c). To determine whether a change in neuronal numbers in the locus coeruleus translated to more dopaminergic input onto the CA1 hippocampus, an area known for receiving large amounts of projections from the locus coeruleus [41], we quantified the amount of TH^+^ fibres in the whole hippocampus and found significantly more of these tracts in the CA1 region in Y^+^-O mice (Fig. 3d; Additional file 4: Figure S4a, b).

### Young Sca-1^+^ stem cells improve hippocampal LTP in aged brains after irradiation

**Fig. 4:**
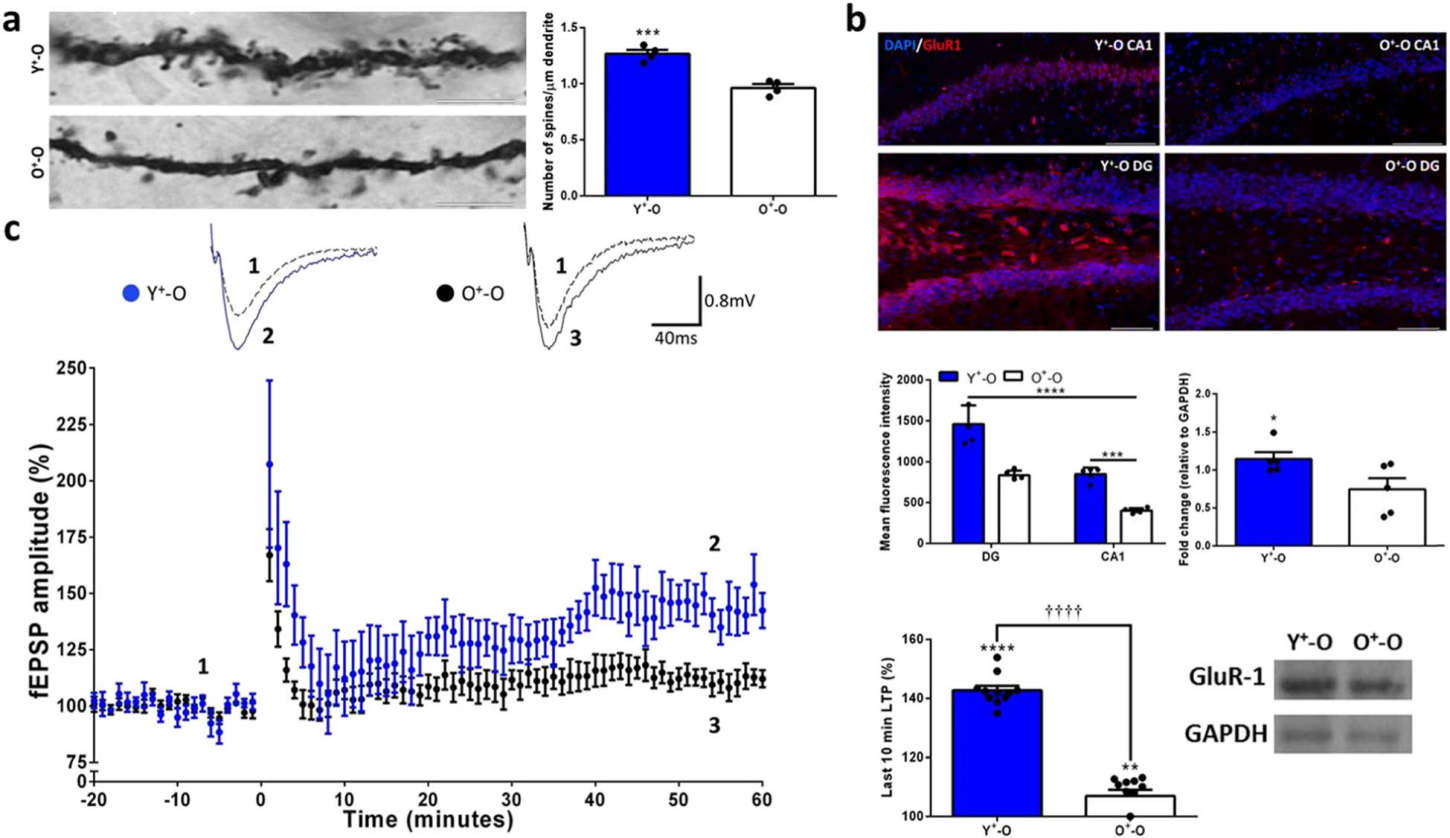
Young Sca-1^+^ stem cells preserve synaptic connectivity and improve LTP in the CA1 hippocampus. **a** Representative image of dendritic spines from CA1 pyramidal neurons using Golgi-Cox staining for *n* = 4 mice per group. **b** Immunostaining for glutamatergic neurons in the CA1 hippocampus and dentate gyrus (top) and representative western-blot analysis for glutamate receptors (GluR1) from the whole hippocampus for at least *n* = 5 mice per group (bottom). **c** Field excitatory postsynaptic potential recordings and quantification from the CA1 hippocampus for at least *n* = 7 hippocampal slices per group. Scale bars, 5 μm (**a**), 200 μm and 100 μm for top and bottom images, respectively (**b**). Data are mean ± s.e.m. *P ≤ 0.05; **P ≤ 0.01; ***P ≤ 0.001;†††† (after stimulation) or **** (relative to baseline before stimulation) *P* ≤ 0.0001 (two-way ANOVA with Tukey’s multiple comparisons test (**b** (left; F (1, 16) = 93.06, *P* < 0.0001)) and unpaired two-sided *t*-tests (**a**, **b** (right), **c**))

In order to investigate whether dopaminergic input to the hippocampus improved synaptic connections within this region, we examined synaptic properties of CA1 pyramidal cells and found the number of dendritic spines in the Y^+^-O group is significantly higher than that in the O^+^-O group (Fig. 4a). Moreover, we also found significantly more glutamate receptors in the dentate gyrus and CA1 region of the hippocampus in Y^+^-O mice (Fig. 4b, Additional file 5: Figure S5a). To determine whether this has an impact on hippocampal function, we recorded excitatory postsynaptic field potentials in the CA1 hippocampus and found significantly greater LTP in Y^+^-O mice, whereas basal synaptic transmission and presynaptic short-term plasticity (i.e., input-output relationship and pair-pulse ratio) were unaffected when compared to O^+^-O mice (Fig. 4c; Additional file 6: Figure S6a, b).

### Young and old BM-derived Iba1^+^ cells are differentially activated in the aged brain

**Fig. 5:**
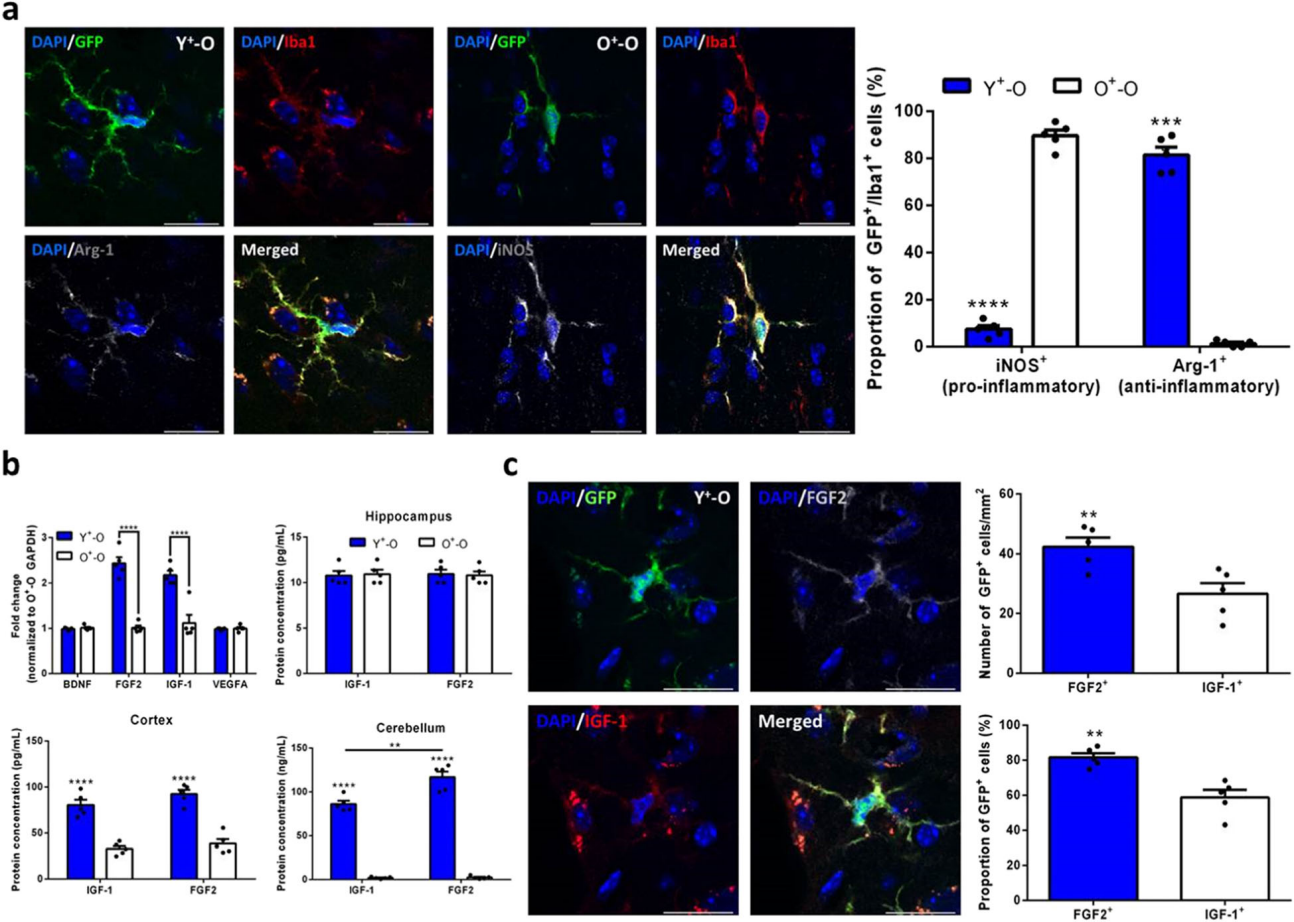
Young and old BM-derived microglia are differentially polarized in the aged brain. **a** Immunostaining and quantification for anti- and pro-inflammatory markers Arg-1 and iNOS, respectively. *n* = 5 mice per group. **b** RTqPCR on whole brain and ELISA on different brain regions for common soluble factors produced by Arg-1^+^ microglia. **c** Immunostaining and quantification of GFP^+^ microglia positive for insulin-like growth factor 1 (IGF-1) and fibroblast growth factor 2 (FGF2) in Y^+^-O brains for *n* = 5 mice per group. Scale bar, 40 μm (**a**, **b**). Data are mean ± s.e.m. **P ≤ 0.01; ***P ≤ 0.001; ****P ≤ 0.0001 (two-way ANOVA with Tukey’s multiple comparisons test (**a** (F (1, 16) = 10.33, *P* = 0.0054), **b** (top left; F (1, 32) = 193.9, *P* < 0.0001, top right; F (1, 16) = 4.068e^−0.005^, *P* = 0.9950, bottom left; F (1, 16) = 118.1, *P* < 0.0001, bottom right; F (1, 16) = 703.6, *P* < 0.0001)) and unpaired two-sided *t*-test (**c**))

In order to gain mechanistic insight into the beneficial properties of young Iba1^+^ cells, we further categorized our BM-derived Iba1^+^ cells into anti- and pro-inflammatory polarized states based on expression of either arginase-1 (Arg-1) or inducible nitric oxide synthase (iNOS), respectively. We found that GFP^+^/Iba1^+^ cells from old donor BM were primarily iNOS^+^, while GFP^+^/Iba1^+^ cells from young donor BM were exclusively Arg-1^+^ (Fig. 5a). Moreover, we noticed that host microglia were similarly Arg-1^+^ when in the proximity to young BM-derived GFP^+^/Arg-1^+^ cells, possibly suggesting that in addition to influencing host cells, young donor Iba1^+^ cells can also likely affect the aged microenvironment (Additional file 7: Figure S7a, b).

Since nitric oxide is a potent activator of DNA damage-induced apoptosis [42], we next examined cellular proliferation and programmed cell death around the fourth ventricle and locus coeruleus; however, we were unable to detect any significant differences between the two groups (Additional file 8: Figure S8a). This suggested that the neuronal differences seen in the locus coeruleus may be due to neuroprotective effects of microglia. Indeed, we screened for several soluble factors with protective properties that are known to be secreted by Arg-1^+^ microglia and found significantly higher RNA and protein levels of fibroblast growth factor 2 and insulin-like growth factor 1 in Y^+^-O brains (Fig. 5b, c). Taken together, these findings suggest that young BM-derived Iba1^+^ cells possess protective properties that likely stem from paracrine signalling of pro-survival factors.

### Human CD34^+^ HSCs migrate from the BM to the brain of NSG mice following irradiation

Lastly, in order to test the possibility that human BM stem cells can produce cells which migrate to the brain after radiotherapy, we generated a humanized mouse model reconstituted with human CD34^+^ HSCs using immunodeficient nod-scid gamma (NSG) mice for enhanced engraftment of human cells into a mouse BM microenvironment. Approximately 3 months following irradiation and stem cell transplantation, we were able to detect human cells (Ku-80^+^ nuclei) in various different brain regions, including the hippocampus. Nearly all of these cells were positive for the CD45 hematopoietic cell marker, and in some cases, Ku-80^+^ cells displayed characteristic microglial morphology (Additional file 9: Figure S9a). Although we did not test the behaviour of these animals following transplantation with human CD34^+^ HSCs, these findings highlight the possibility of using human BM-derived HSCs to potentially treat and repair a wide range of neurological disorders, primarily those whose treatments rely on long-term passage across the blood-brain barrier.

## Discussion

Treatments for the progression of cognitive decline following brain irradiation have been met with some challenges. Pharmaceutical approaches have been limited to animal models and carried out using low doses of radiation for short-term periods [43]. Intracerebral transplantations of human embryonic or neural stem cells provide some benefit in cognitive recovery; however, limited therapeutic potential has been observed due to minimal cell engraftment, low survival of implanted cells in long-term therapy, and the invasive nature of treatment [44, 45]. In this study, we present a minimally invasive approach with long-term benefits in aged recipients. We demonstrate that the intravenously injected, self-renewing population of young HSCs, are able to successfully repopulate the BM and migrate to the brain in large numbers. In brain tissue, these GFP^+^/Sca-1^+^ BM cells differentiate into anti-inflammatory Iba1^+^ cells for up to 3 months post-reconstitution. Only in the absence of pro-inflammatory Iba1^+^ cells, which become polarized to this state if they are derived from old Sca-1^+^ stem cells, do the animals display significant long-term improvements in hippocampal-dependent learning and memory following irradiation.

The introduction of stem cells to the brain has been investigated for several decades due to the unique characteristics of the blood-brain barrier. Through the use of heterochronic parabiosis, several circulating blood-borne factors from young animals have been discovered and shown to exhibit beneficial effects on cognitive abilities in aged animals [46]. Despite this, temporarily joining circulatory systems between animals in order to introduce beneficial factors into the brain was found to be limited due to low levels of peripheral myeloid cells capable of crossing the blood-brain barrier [47]. Although friendlier alternatives have been proposed for permeabilizing the blood-brain barrier, such as through ultrasound microbubble destruction for temporary opening [48] or by the use of chemotherapeutic agents [49], radiation to date remains the best tool for achieving the greatest BM chimerism and subsequent engraftment of injected cells into the brain. Here we report a significantly larger amount of human and mouse BM-derived cells residing in the brain than previously documented [50, 51]. This may be due to our administrated radiation dose, which has been shown to be directly proportional to the number of BM cells that engraft in the brain [52]. Another possible mechanism for successful passage of BM cells to the brain may be due to Sca-1 protein expression on the surface of transplanted HSCs, as recently, the GPI-anchored Sca-1 protein has been described to be substantially involved in driving transport across the blood-brain barrier [53].

Despite its many uses, the use of radiation therapy has several drawbacks [54–56]. In this study, we carried out whole-body irradiation at 10 Gy to induce both cognitive impairments and depletion of BM HSCs [57, 58]. Our approach regarding the method by which the radiation was induced has important clinical relevance as patients receiving BM transplant therapy commonly undergo whole-body irradiation [59]. Furthermore, although the radiation dose used in our study is enough to induce long-term cognitive impairments in rodents, radiation doses used in human patients against abnormal cell growth in the brain are significantly larger and depend on tumour size and recipient age [60, 61]. In cases where whole-body irradiation is required, patients are typically exposed to several regiments of 10 Gy spaced out over multiple days [62]. It has been reported that several fractionated doses of radiation for BM transplantation are sufficient to induce long-term cognitive decline in up to 60% of surviving patients [63–66]. Despite the high incidence of cognitive complications following whole-body irradiation, modern practice has slowly been shifting towards protecting major organs from radiation-induced injury, such as the lungs [67], although shielding the brain has yet to be implemented. As such, our model presented herein has strong clinical significance for ameliorating primary and secondary induced cognitive decline following focal brain and whole-body radiotherapy for brain cancer and BM transplantation, respectively.

Recovery of radiotherapy-induced loss of cognitive capacity for the treatment of brain malignancies in the elderly population is limited [68]. Although the direct cause of this remains to be known, declining numbers of BM stem cell pools in geriatric animals have been correlated with poor functional outcomes following injury [69–71]. In the current study, we address the issue of the limited regenerative capacity in aged animals by repopulating the aged BM with an enriched population of young Sca-1^+^ stem cells that are potentially capable of crossing the blood-brain barrier. However, the role of BM-derived cells in blood-brain barrier-devoid regions has yet to be investigated. Since we and others [72–74] report a large number of GFP^+^ cells in contact with ependymal cells of the choroid plexus, it would be interesting to investigate whether any beneficial circulating factors are secreted into the cerebrospinal fluid by these cells post-reconstitution. Indeed, the role of BM-derived cells in semi-permeable regions of the blood-brain barrier is an alternative direction of treatment as it does not require disturbing endothelial cells lining the blood vessels.

It is known that cognitive abilities are highly correlated with microglial function, and recently, microglial roles and functions of different polarization/activation states during brain development and degenerative progress have received great attention. Little is known about their contribution(s) towards neuronal function in young and aged brain tissue. Recently, it was reported that BM cells are able to mobilize to brain tissue, where they become microglia and reduce circulating CCL11 cytokine levels (when using young, but not old, BM), leading to preserved learning and memory in old mice [75]. In this study, we reported that young and old HSCs give rise to differentially activated Iba1^+^ cells upon exposure to identical signals within the aged brain microenvironment. We found that young BM-derived Iba1^+^ cells acquire an anti-inflammatory profile in the aged brain (Arg-1^+^), whereas old BM-derived Iba1^+^ cells differentiate into cells approximating the identity of aged host microglia (iNOS^+^). These results suggest that young cells possess some form of resistance against inflammatory signals known to be associated with the aged brain milieu. Indeed, Arg-1 is an enzyme that directly competes with iNOS for the same substrate, l-arginine, and can thus act to downregulate nitric oxide production [76]. This phenomenon provides an explanation for our findings that aged host microglia (Iba1^+^/GFP^−^) acquire an anti-inflammatory polarized state only when near the vicinity of young BM-derived anti-inflammatory Iba1^+^ cells. This novel finding provides a new direction for treating and reversing age- and radiation-induced cognitive deficits. Past studies have already demonstrated that complete depletion of brain microglia is not a terminal procedure and that the lost microglia can repopulate from a combination of nestin^+^ non-microglial progenitor cells (i.e., non-BM-derived progenitor cells) and surviving microglia following depletion [14, 17]. Whichever the case may be, the newly formed microglia which arise from aged cells may be vulnerable to signals originating from the aged brain microenvironment which trigger the adoption of a pro-inflammatory phenotype. To address this issue, repopulation of ablated aged microglia from young BM-derived stem and progenitor cells following blood-brain barrier permeabilization may provide a long-term solution for strengthening cognitive abilities and regenerative potential of aged brains. Indeed, a recent study showed that selectively targeting and inhibiting microglial pro-inflammatory cytokine production following a high dose of radiation mitigated radiation-induced cognitive impairments [77].

Despite the positive effects exerted on cognitive capacity following injection of young Sca-1^+^ stem cells, one of the limitations of this study is not examining the potential interplay between donor stem cells and hyperplasia. Indeed, our array data reveals that young Sca-1^+^ stem cells upregulate several genes related to development, cytoskeleton reorganization, and induction of growth factors. Although it remains unclear whether healthy young BM stem cells could directly contribute to tumour formation, BM stem cells have been previously documented in promoting the growth of existing cancer cells [78, 79]. Moreover, processes such as cytoskeleton remodelling have been implicated in malignant cell metastasis and tumour formation [80, 81]. However, despite the increased incidence of tumourigenesis with age [82], aged recipients of young Sca-1^+^ stem cells were negative for any noticeable abnormal masses. This may be due to newly transplanted young BM cells possessing natural resistant to negative influence from the aged microenvironment, similar to what is described for BM-derived microglial-like cells in the brain. This is important to consider in cases where the BM niche is the primary target and origin for blood cancer and related disorders that may potentially and ultimately lead to global downstream consequences [83].

## Conclusion

Collectively, these results indicate that the intravenously injected, self-renewing young HSCs, are able to repopulate the BM and migrate to the brain where they then differentiate into anti-inflammatory microglia for up to 3 months post-reconstitution. Only in the absence of pro-inflammatory microglia, which become polarized to this state if they are derived from old Sca-1^+^ stem cells, do the animals display significant long-term improvements in hippocampal-dependent learning and memory following irradiation. Therefore, we conclude that the introduction of Sca-1^+^ HSCs from young donor BM may provide a novel therapeutic avenue for the treatment of cognitive deficits that arise after radiotherapy of the aged brain.

## Additional files

Additional file 1: **Figure S1.***GFP*^*+*^*cell distribution in the hindbrain (cerebellum) with occasional functional GFP*^*+*^*Purkinje cells*. (a) GFP^+^ cells and commonly observed morphologies (insets) in the hindbrain of an O^+^-O animal. (b) GFP^+^ cells in the cerebellum of a single O^+^-O animal with an enlarged image of a GFP^+^ Purkinje cell and its recorded action potential. Arrow points to a second GFP^+^ Purkinje cell and arrowhead depicts a GFP^+^ microglia. Scale bars, from left to right (a): 2 mm, 100 μm and 10 μm; (b): 300 μm, 25 μm and 10 μm. (DOCX 1682 kb)

Additional file 2: **Figure S2.***Number of pro-inflammatory microglia increase with age while spatial memory and learning performance declines*. (a) Representative immunostaining and quantification of pro-inflammatory (iNOS^+^) Iba1^+^ microglia in 2-month-old and 18-month-old WT mice. *n* = 6 mice per group. (b) Immunostaining and quantification of WT microglia morphology with aging. *n* = 6 mice per group. (c) Spatial memory Barnes maze (top) and novel object recognition (bottom) performance for 2-month-old and 18-month-old WT mice. *n* = 6 mice per group. Scale bars 100 μm (a) and 10 μm (b). Data are mean ± s.e.m. *P ≤ 0.05; **P ≤ 0.01; ***P ≤ 0.001; ****P ≤ 0.0001 (two-way ANOVA with Tukey’s multiple comparisons test (c top; F (1, 31) = 22.52, *P* < 0.0001, bottom; F (1, 20) = 160.2, *P* < 0.0001) and unpaired two-sided t-tests (a, b)) (DOCX 1104 kb)

Additional file 3: **Figure S3.*** Genetic changes in the whole brain of old mice reconstituted with young Sca-1*^*+*^*or Sca-1*^*−*^*cells*. (a) Hierarchical clustering on gene level counts of differentially expressed genes as a function of experimental treatment for *n* = 3 mice per groups (FC ≥ 1.5, *P* ≤ 0.05). (b) Volcano plot of 2056 significantly dysregulated genes and (c) a STRING interaction network for the norepinephrine transporter gene, SLC6A2, depicted by the black arrow (right). (DOCX 128 kb)

Additional file 4: **Figure S4.*** Tyrosine hydroxylase expression in the reconstituted hippocampus*. (a) Western-blot analysis and quantification in the whole hippocampus for *n* = 5 animals per group. (b) Immunostaining and quantification for tyrosine hydroxylase fibers in different regions of the hippocampus for *n* = 5 animals per group. Scale bars, 200 μm (top and middle panels) and 100 μm for bottom panels (a). Data are mean ± s.e.m. *P ≤ 0.05; ****P ≤ 0.0001 (unpaired two-sided t-tests (a) and two-way ANOVA with Tukey’s multiple comparisons test (b F (2, 24) = 587.4, *P* < 0.0001)). (DOCX 134 kb)

Additional file 5: **Figure S5.***PSD-95 and glutamatergic neuron levels in the reconstituted hippocampus*. (a) Western-blot analysis and quantification in the whole hippocampus for *n* = 5 animals per group. Data are mean ± s.e.m. *P ≤ 0.05 (unpaired two-sided t-tests (a)). (DOCX 73 kb)

Additional file 6: **Figure S6.*** Basal synaptic transmission and presynaptic function in the CA1 hippocampus*. (a) Input-output curves for *n* = 4 hippocampal slices per group. (b) Paired-pulse facilitation for at least *n* = 7 hippocampal slices per group. (DOCX 174 kb)

Additional file 7: **Figure S7.***Young donor microglia can influence the polarized state of old host microglia*. (a) Immunostaining for anti-inflammatory (Arg-1^+^) donor (GFP^+^) and host (GFP^−^) microglia in a midbrain section containing the hippocampus. (b) Higher magnification inset from a Y^+^-O mouse demonstrating signal localization and cell proximity (left) and quantification of immunostaining (right). *n* = 5 mice per group for five randomly selected regions. Scale bars 100 μm (a) and 50 μm for inset (b). Data are mean ± s.e.m. ****P ≤ 0.0001 (unpaired two-sided t-tests (b)). (DOCX 198 kb)

Additional file 8: **Figure S8.***Apoptosis and cell proliferation around the locus coeruleus and fourth ventricle*. (a) Immunostaining and quantification of proliferating Ki-67^+^ and apoptotic TUNEL^+^ nuclei (shown in red). *n* = 5 mice per group. Scale bar, 200 μm (a). Data are mean ± s.e.m. (unpaired two-sided t-tests (a)). (DOCX 117 kb)

Additional file 9: **Figure S9.***Reconstituted human CD34*^*+*^*HSCs migrate from the BM to the brain.* (a) Immunostaining and quantification of human Ku-80^+^/CD45^+^ cells in various different regions of the mouse brain. *n* = 5 mice. Scale bar, 200 μm (a). Data are mean ± s.e.m. (one-way ANOVA with Tukey’s multiple comparisons test tests (a)). (DOCX 121 kb)

## Data Availability

The datasets used and/or analyzed during the current study are available from the corresponding author on reasonable request.

## Abbreviations

Arg-1: Arginase-1
BM: Bone marrow
FGF2: Fibroblast growth factor 2
GFP: Green fluorescent protein
GluR1: Glutamate receptor
HSCs: Hematopoietic stem cells
IGF-1: Insulin-like growth factor-1
iNOS: Inducible nitric oxide synthase
LTP: Long-term potentiation
PSD95: Postsynaptic density protein 95
Sca-1: Stem cell antigen-1
TH: Tyrosine hydroxylase
