# Supplementary material for: Rectification of radiotherapy-induced cognitive impairments in aged mice by reconstituted Sca-1+ stem cells from young donors
Source: J Neuroinflammation. 2020 Feb 7;17:51. doi: 10.1186/s12974-019-1681-3 (PMC7006105; doi:10.1186/s12974-019-1681-3)
Supplement: Supplementary file 1 — Figure S1.GFP+cell distribution in the hindbrain (cerebellum) with occasional functional GFP+Purkinje cells. (a) GFP+ cells and commonly observed morphologies (insets) in the hindbrain of an O+-O animal. (b) GFP+ cells in the cerebellum of a single O+-O animal with an enlarged image of a GFP+ Purkinje cell and its recorded action potential. Arrow points to a second GFP+ Purkinje cell and arrowhead depicts a GFP+ microglia. Scale bars, from left to right (a): 2 mm, 100 μm and 10 μm; (b): 300 μm, 25 μm and 10 μm. (DOCX 1682 kb) [file 12974_2019_1681_MOESM1_ESM.docx]

**
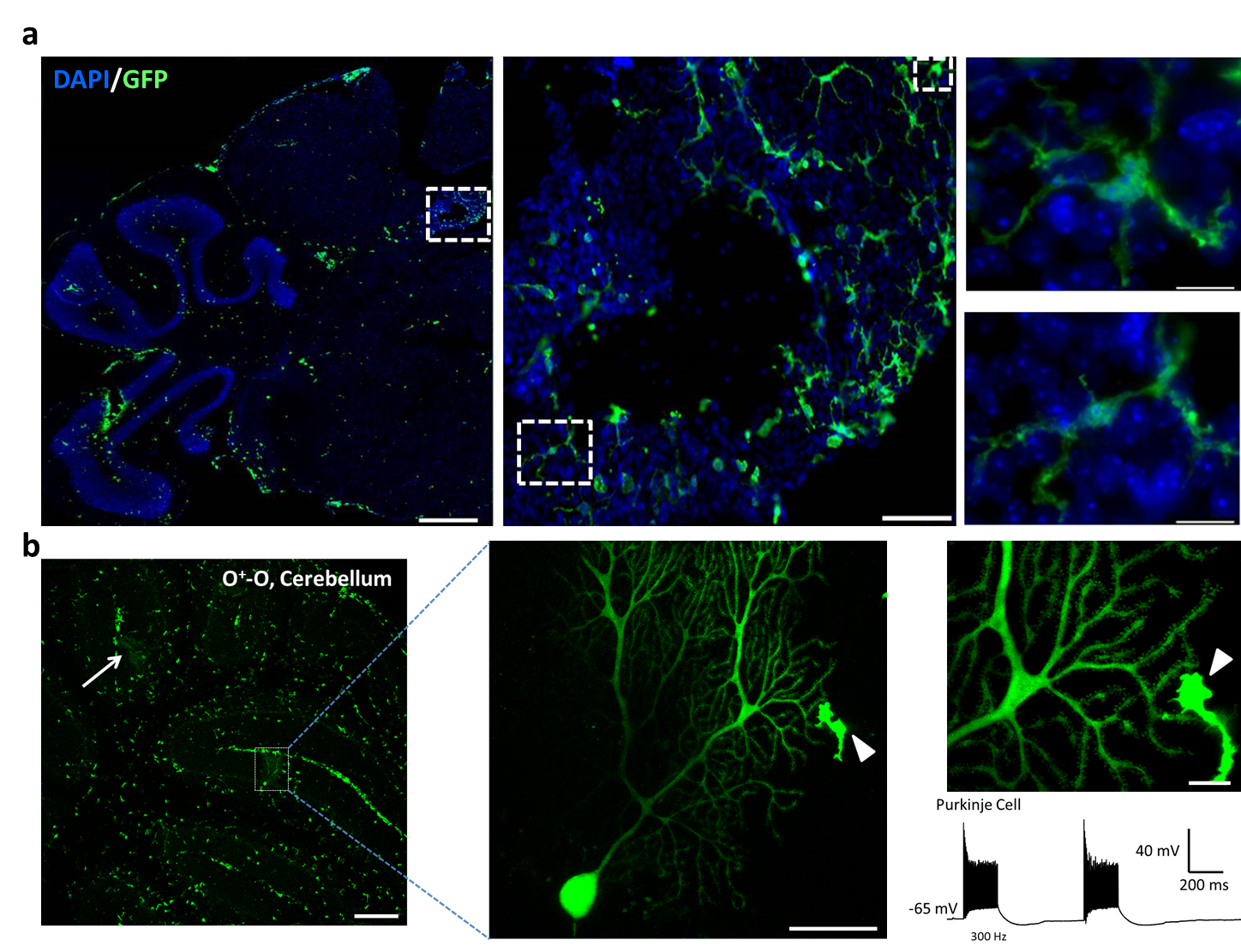
**

*Figure S1: GFP^+^ cell distribution in the hindbrain (cerebellum) with occasional functional GFP^+^ Purkinje cells*. (a) GFP^+^ cells and commonly observed morphologies (insets) in the hindbrain of an O^+^-O animal. (b) GFP^+^ cells in the cerebellum of a single O^+^-O animal with an enlarged image of a GFP^+^ Purkinje cell and its recorded action potential. Arrow points to a second GFP^+^ Purkinje cell and arrowhead depicts a GFP^+^ microglia. Scale bars, from left to right (a): 2 mm, 100 µm and 10 µm; (b): 300 µm, 25 µm and 10 µm.
