# Supplementary material for: Rectification of radiotherapy-induced cognitive impairments in aged mice by reconstituted Sca-1+ stem cells from young donors
Source: J Neuroinflammation. 2020 Feb 7;17:51. doi: 10.1186/s12974-019-1681-3 (PMC7006105; doi:10.1186/s12974-019-1681-3)
Supplement: Supplementary file 2 — Figure S2.Number of pro-inflammatory microglia increase with age while spatial memory and learning performance declines. (a) Representative immunostaining and quantification of pro-inflammatory (iNOS+) Iba1+ microglia in 2-month-old and 18-month-old WT mice. n = 6 mice per group. (b) Immunostaining and quantification of WT microglia morphology with aging. n = 6 mice per group. (c) Spatial memory Barnes maze (top) and novel object recognition (bottom) performance for 2-month-old and 18-month-old WT mice. n = 6 mice per group. Scale bars 100 μm (a) and 10 μm (b). Data are mean ± s.e.m. *P ≤ 0.05; **P ≤ 0.01; ***P ≤ 0.001; ****P ≤ 0.0001 (two-way ANOVA with Tukey’s multiple comparisons test (c top; F (1, 31) = 22.52, P < 0.0001, bottom; F (1, 20) = 160.2, P < 0.0001) and unpaired two-sided t-tests (a, b)) (DOCX 1104 kb) [file 12974_2019_1681_MOESM2_ESM.docx]

**
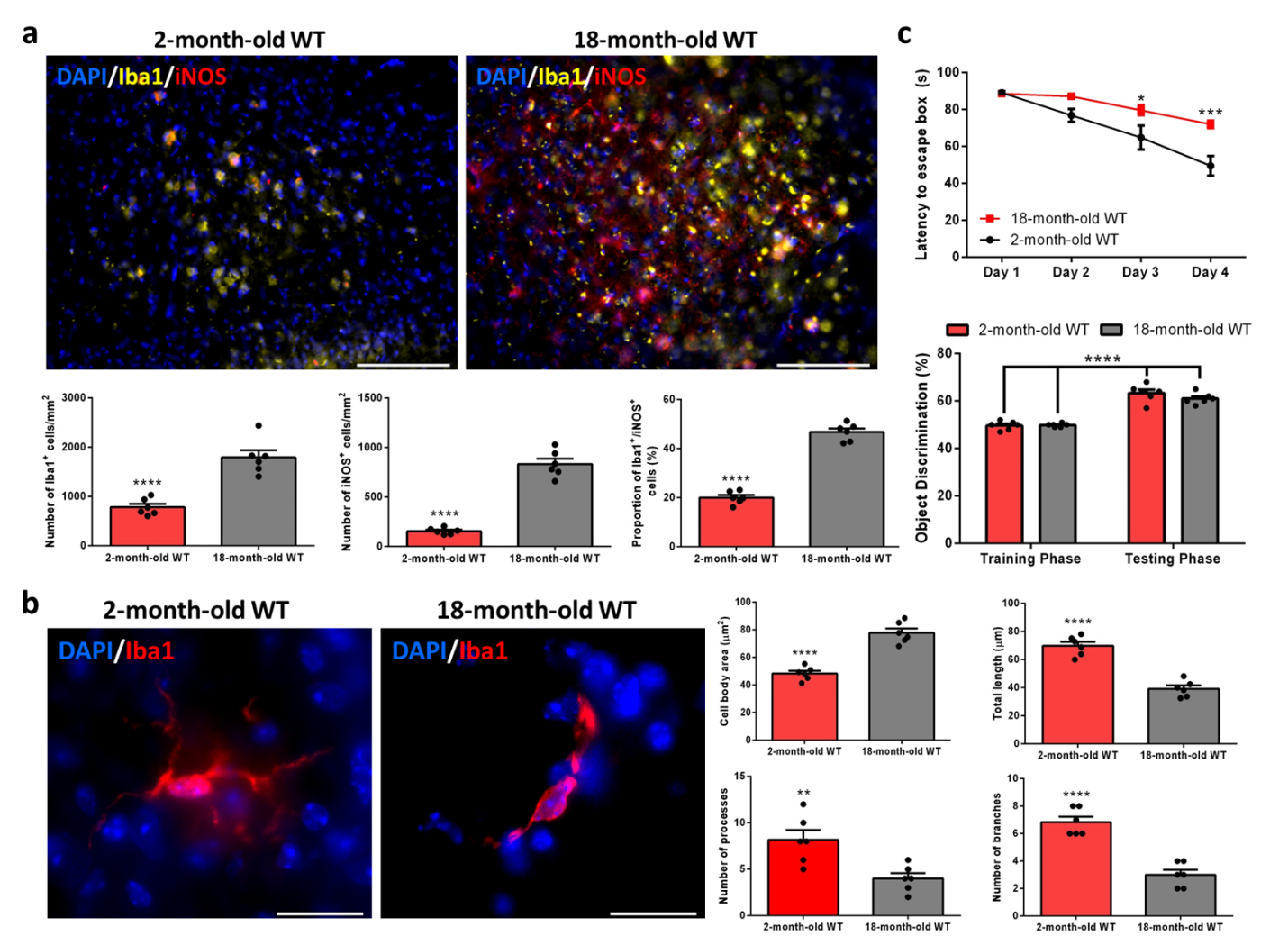
**

*Figure S2: Number of pro-inflammatory microglia increase with age while spatial memory and learning performance declines*. (a) Representative immunostaining and quantification of pro-inflammatory (iNOS^+^) Iba1^+^ microglia in 2-month-old and 18-month-old WT mice. *n* = 6 mice per group. (b) Immunostaining and quantification of WT microglia morphology with aging. *n* = 6 mice per group. (c) Spatial memory Barnes maze (top) and novel object recognition (bottom) performance for 2-month-old and 18-month-old WT mice. *n* = 6 mice per group. Scale bars 100 µm (a) and 10 µm (b). Data are mean ± s.e.m. **P* ≤ 0.05; ***P* ≤ 0.01; ****P* ≤ 0.001; *****P* ≤ 0.0001 (two-way ANOVA with Tukey's multiple comparisons test (c top; F (1, 40) = 22.52, *P* < 0.0001, bottom; F (1, 20) = 160.2, *P* < 0.0001) and unpaired two-sided t-tests (a, b)).
