# Supplementary material for: Rectification of radiotherapy-induced cognitive impairments in aged mice by reconstituted Sca-1+ stem cells from young donors
Source: J Neuroinflammation. 2020 Feb 7;17:51. doi: 10.1186/s12974-019-1681-3 (PMC7006105; doi:10.1186/s12974-019-1681-3)
Supplement: Supplementary file 3 — Figure S3. Genetic changes in the whole brain of old mice reconstituted with young Sca-1+or Sca-1−cells. (a) Hierarchical clustering on gene level counts of differentially expressed genes as a function of experimental treatment for n = 3 mice per groups (FC ≥ 1.5, P ≤ 0.05). (b) Volcano plot of 2056 significantly dysregulated genes and (c) a STRING interaction network for the norepinephrine transporter gene, SLC6A2, depicted by the black arrow (right). (DOCX 128 kb) [file 12974_2019_1681_MOESM3_ESM.docx]

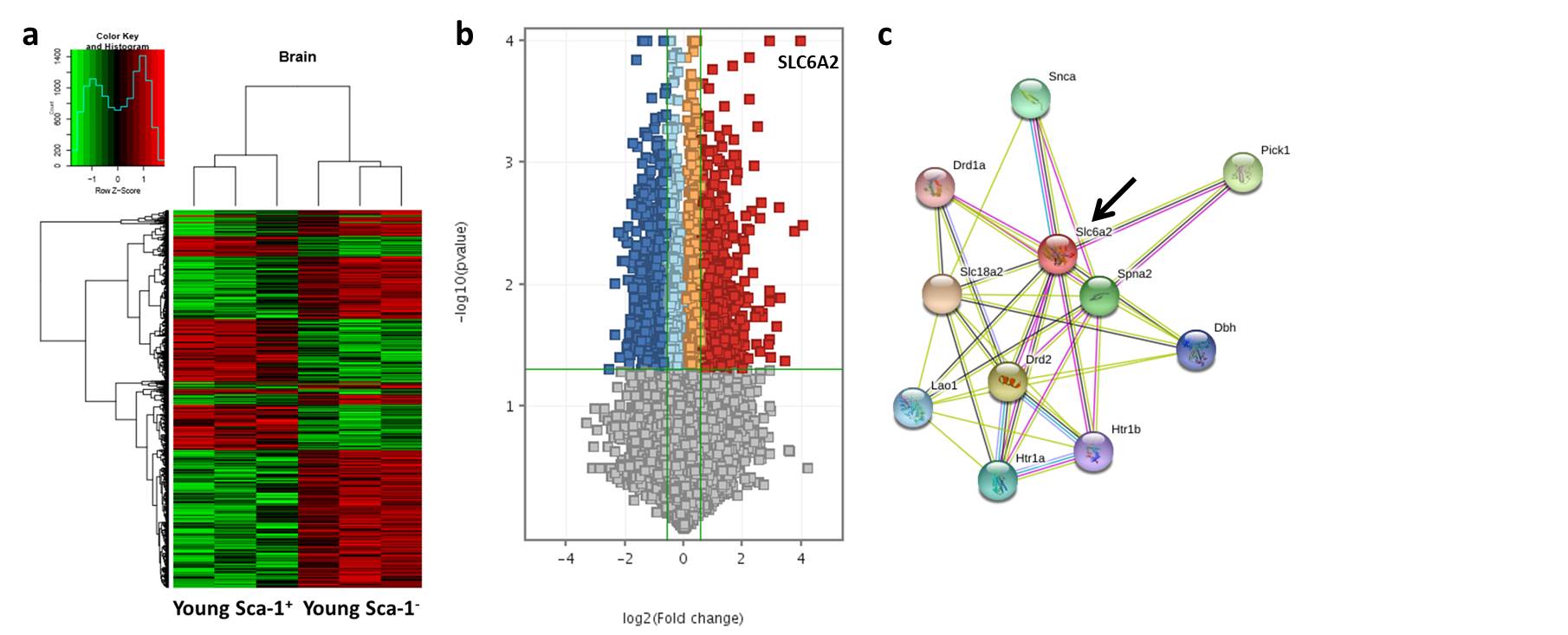


*Figure S3: Genetic changes in the whole brain of old mice reconstituted with young Sca-1^+^ or Sca-1^-^ cells*. (a) Hierarchical clustering on gene level counts of differentially expressed genes as a function of experimental treatment for *n* = 3 mice per groups (FC ≥ 1.5, *P* ≤ 0.05). (b) Volcano plot of 2056 significantly dysregulated genes and (c) a STRING interaction network for the norepinephrine transporter gene, SLC6A2, depicted by the black arrow (right).
