# Supplementary material for: Rectification of radiotherapy-induced cognitive impairments in aged mice by reconstituted Sca-1+ stem cells from young donors
Source: J Neuroinflammation. 2020 Feb 7;17:51. doi: 10.1186/s12974-019-1681-3 (PMC7006105; doi:10.1186/s12974-019-1681-3)
Supplement: Supplementary file 4 — Figure S4. Tyrosine hydroxylase expression in the reconstituted hippocampus. (a) Western-blot analysis and quantification in the whole hippocampus for n = 5 animals per group. (b) Immunostaining and quantification for tyrosine hydroxylase fibers in different regions of the hippocampus for n = 5 animals per group. Scale bars, 200 μm (top and middle panels) and 100 μm for bottom panels (a). Data are mean ± s.e.m. *P ≤ 0.05; ****P ≤ 0.0001 (unpaired two-sided t-tests (a) and two-way ANOVA with Tukey’s multiple comparisons test (b F (2, 24) = 587.4, P < 0.0001)). (DOCX 134 kb) [file 12974_2019_1681_MOESM4_ESM.docx]

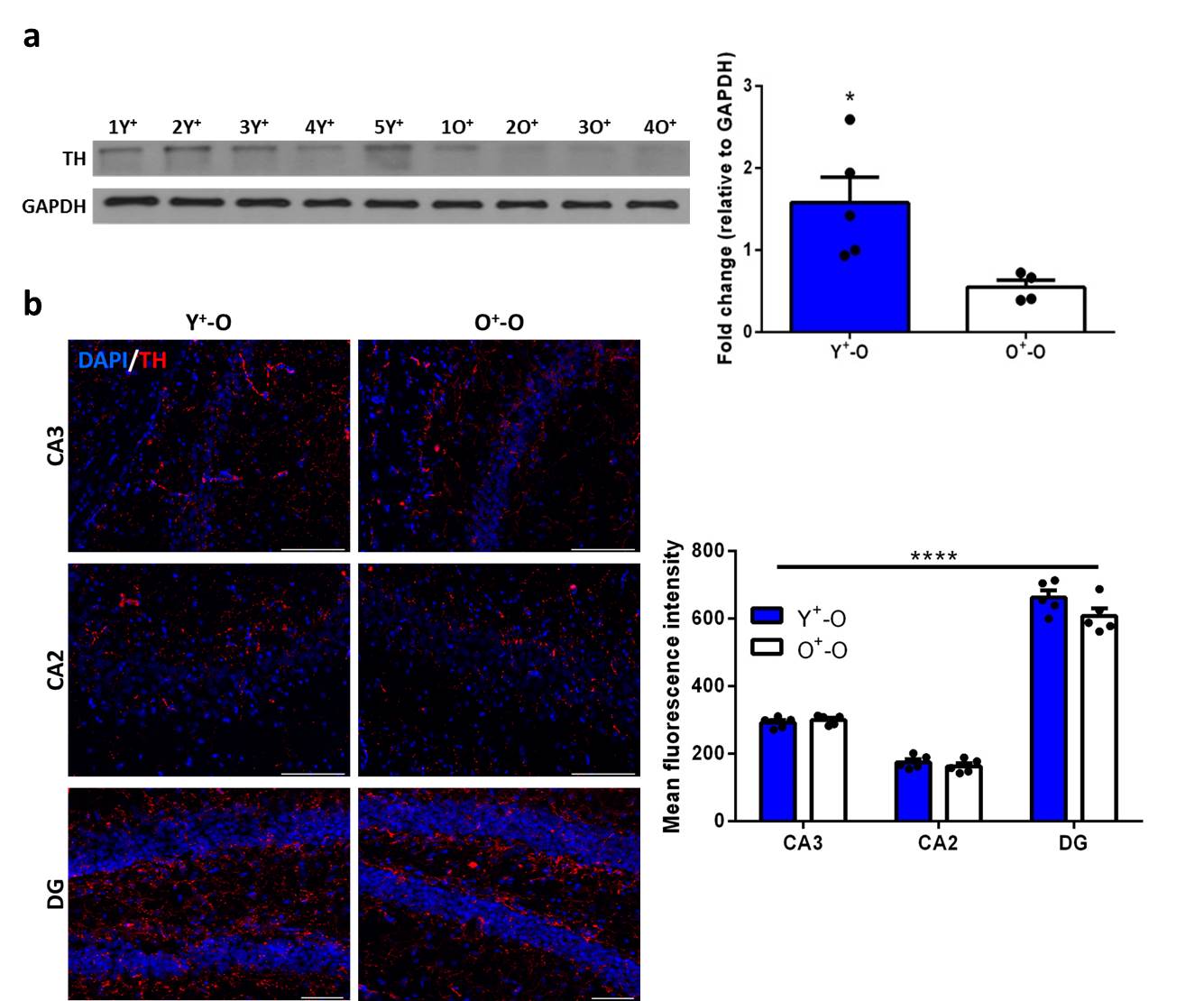


*Figure S4: Tyrosine hydroxylase expression in the reconstituted hippocampus*. (a) Western-blot analysis and quantification in the whole hippocampus for *n* = 5 animals per group. (b) Immunostaining and quantification for tyrosine hydroxylase fibres in different regions of the hippocampus for *n* = 5 animals per group. Scale bars, 200 µm (top and middle panels) and 100 µm for bottom panels (a). Data are mean ± s.e.m. **P* ≤ 0.05; *****P* ≤ 0.0001 (unpaired two-sided t-tests (a) and two-way ANOVA with Tukey's multiple comparisons test (b F (2, 24) = 587.4, *P* < 0.0001)).
