# Supplementary material for: Rectification of radiotherapy-induced cognitive impairments in aged mice by reconstituted Sca-1+ stem cells from young donors
Source: J Neuroinflammation. 2020 Feb 7;17:51. doi: 10.1186/s12974-019-1681-3 (PMC7006105; doi:10.1186/s12974-019-1681-3)
Supplement: Supplementary file 5 — Figure S5.PSD-95 and glutamatergic neuron levels in the reconstituted hippocampus. (a) Western-blot analysis and quantification in the whole hippocampus for n = 5 animals per group. Data are mean ± s.e.m. *P ≤ 0.05 (unpaired two-sided t-tests (a)). (DOCX 73 kb) [file 12974_2019_1681_MOESM5_ESM.docx]

**
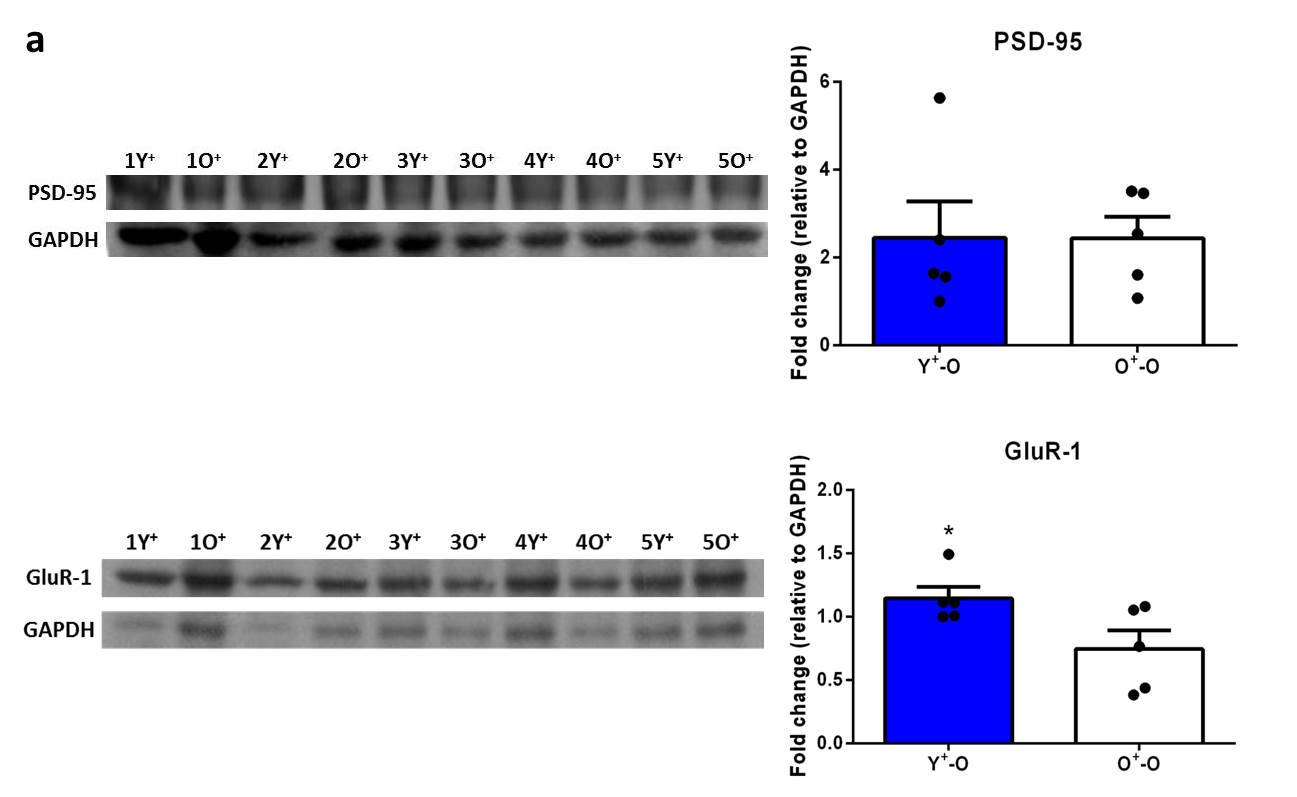
**

*Figure S5: PSD-95 and glutamatergic neuron levels in the reconstituted hippocampus*. (a) Western-blot analysis and quantification in the whole hippocampus for *n* = 5 animals per group. Data are mean ± s.e.m. **P* ≤ 0.05 (unpaired two-sided t-tests (a)).
