# Supplementary material for: Rectification of radiotherapy-induced cognitive impairments in aged mice by reconstituted Sca-1+ stem cells from young donors
Source: J Neuroinflammation. 2020 Feb 7;17:51. doi: 10.1186/s12974-019-1681-3 (PMC7006105; doi:10.1186/s12974-019-1681-3)
Supplement: Supplementary file 6 — Figure S6. Basal synaptic transmission and presynaptic function in the CA1 hippocampus. (a) Input-output curves for n = 4 hippocampal slices per group. (b) Paired-pulse facilitation for at least n = 7 hippocampal slices per group. (DOCX 174 kb) [file 12974_2019_1681_MOESM6_ESM.docx]

*
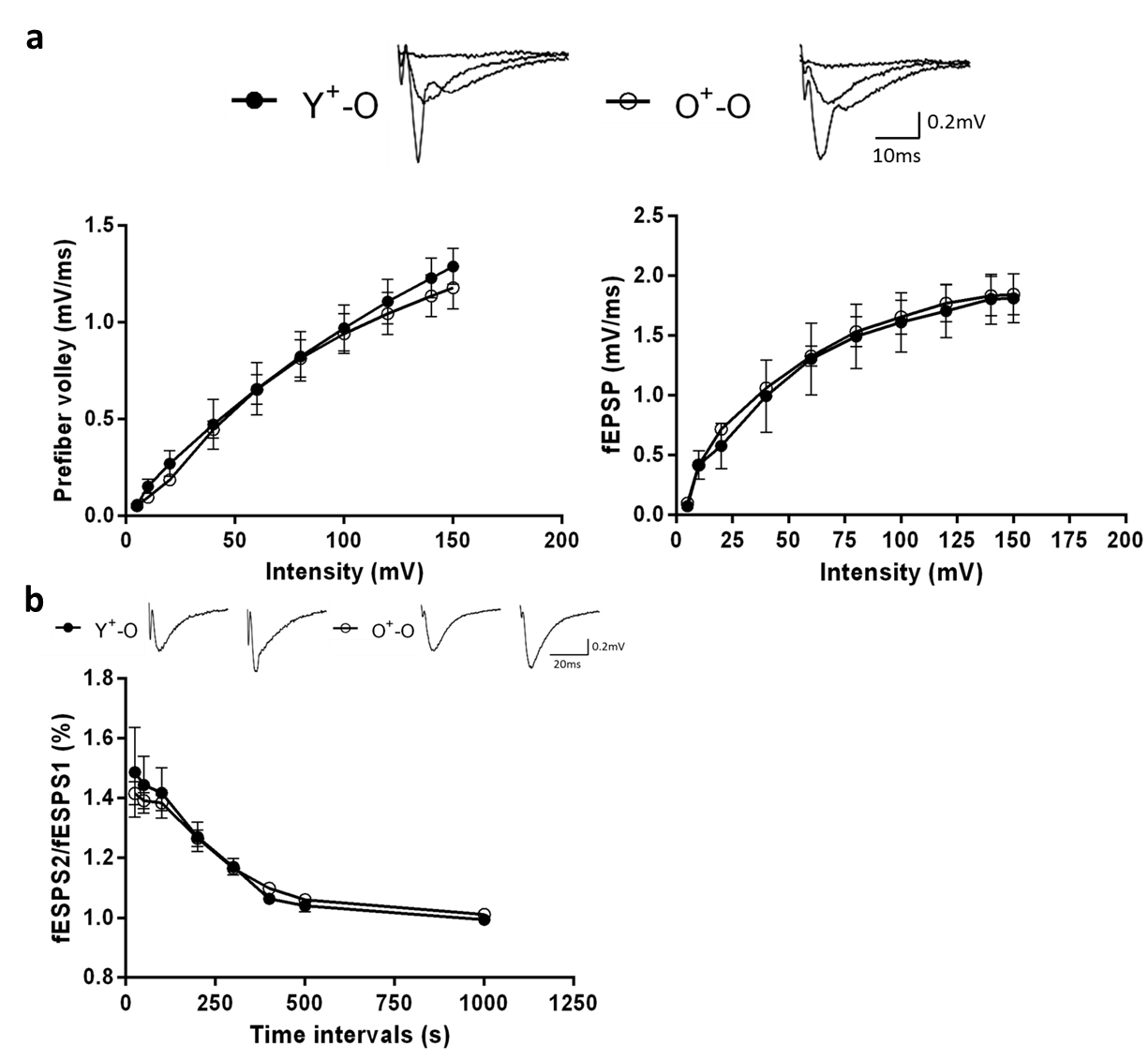
*

*Figure S6: Basal synaptic transmission and presynaptic function in the CA1 hippocampus*. (a) Input-output curves for *n* = 4 hippocampal slices per group. (b) Paired-pulse facilitation for at least *n* = 7 hippocampal slices per group.
