# Supplementary material for: Rectification of radiotherapy-induced cognitive impairments in aged mice by reconstituted Sca-1+ stem cells from young donors
Source: J Neuroinflammation. 2020 Feb 7;17:51. doi: 10.1186/s12974-019-1681-3 (PMC7006105; doi:10.1186/s12974-019-1681-3)
Supplement: Supplementary file 7 — Figure S7.Young donor microglia can influence the polarized state of old host microglia. (a) Immunostaining for anti-inflammatory (Arg-1+) donor (GFP+) and host (GFP−) microglia in a midbrain section containing the hippocampus. (b) Higher magnification inset from a Y+-O mouse demonstrating signal localization and cell proximity (left) and quantification of immunostaining (right). n = 5 mice per group for five randomly selected regions. Scale bars 100 μm (a) and 50 μm for inset (b). Data are mean ± s.e.m. ****P ≤ 0.0001 (unpaired two-sided t-tests (b)). (DOCX 198 kb) [file 12974_2019_1681_MOESM7_ESM.docx]

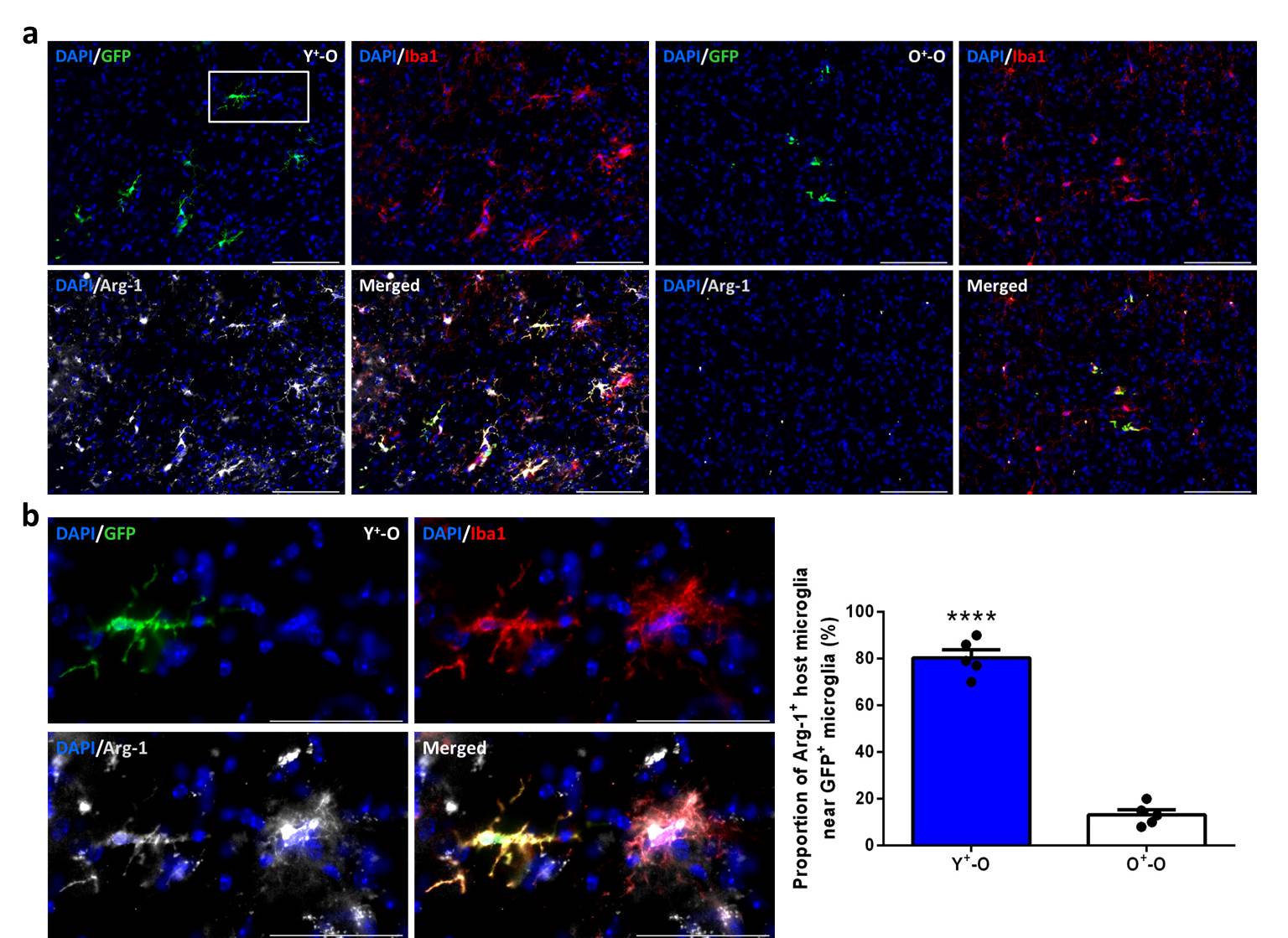


*Figure S7: Young donor microglia can influence the polarized state of old host microglia*. (a) Immunostaining for anti-inflammatory (Arg-1^+^) donor (GFP^+^) and host (GFP^-^) microglia in a midbrain section containing the hippocampus. (b) Higher magnification inset from a Y^+^-O mouse demonstrating signal localization and cell proximity (left) and quantification of immunostaining (right). *n* = 5 mice per group for five randomly selected regions. Scale bars 100 µm (a) and 50 µm for inset (b). Data are mean ± s.e.m. *****P* ≤ 0.0001 (unpaired two-sided t-tests (b)).
