# Supplementary material for: Rectification of radiotherapy-induced cognitive impairments in aged mice by reconstituted Sca-1+ stem cells from young donors
Source: J Neuroinflammation. 2020 Feb 7;17:51. doi: 10.1186/s12974-019-1681-3 (PMC7006105; doi:10.1186/s12974-019-1681-3)
Supplement: Supplementary file 8 — Figure S8.Apoptosis and cell proliferation around the locus coeruleus and fourth ventricle. (a) Immunostaining and quantification of proliferating Ki-67+ and apoptotic TUNEL+ nuclei (shown in red). n = 5 mice per group. Scale bar, 200 μm (a). Data are mean ± s.e.m. (unpaired two-sided t-tests (a)). (DOCX 117 kb) [file 12974_2019_1681_MOESM8_ESM.docx]

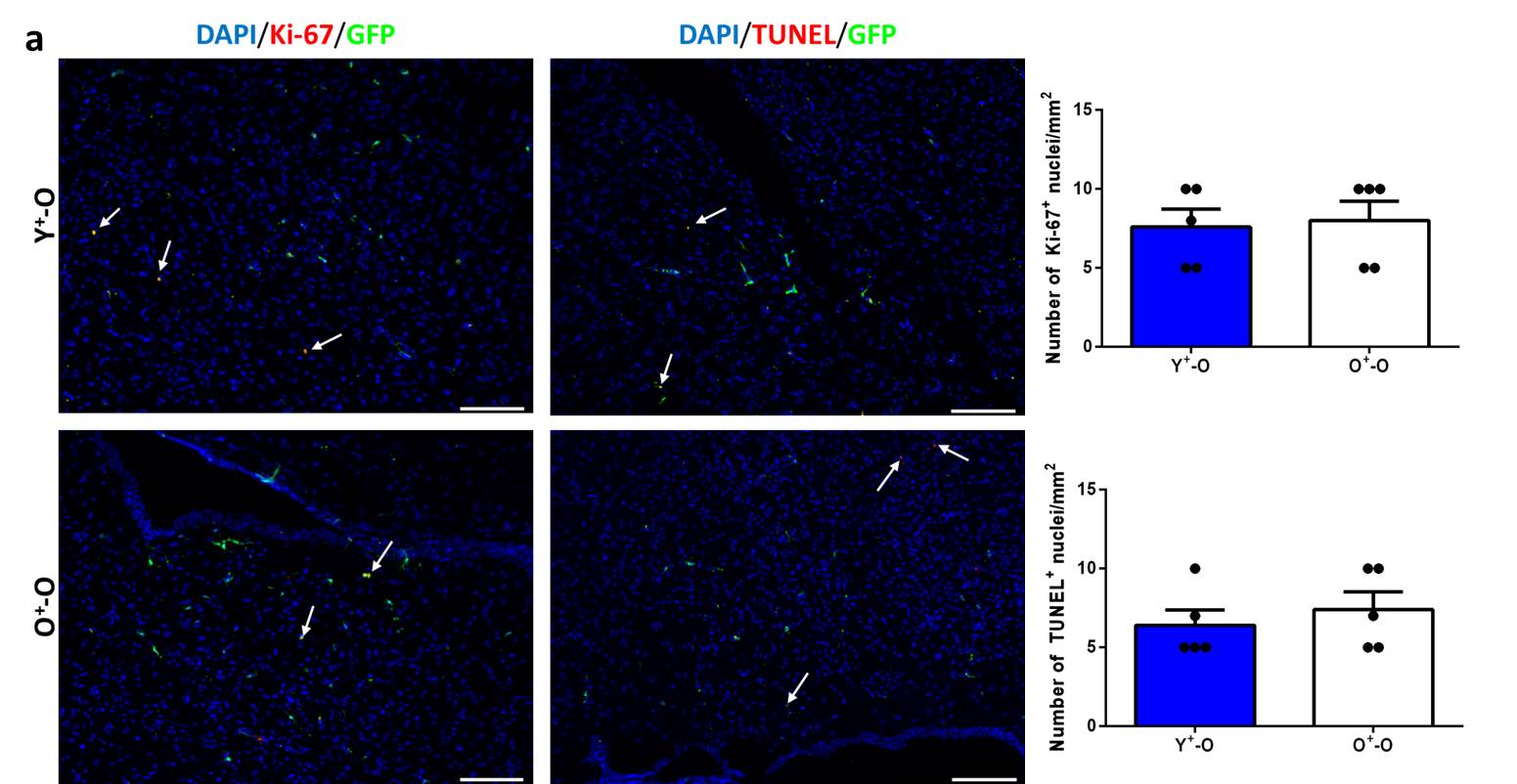


*Figure S8: Apoptosis and cell proliferation around the locus coeruleus and fourth ventricle*. (a) Immunostaining and quantification of proliferating Ki-67^+^ and apoptotic TUNEL^+^ nuclei (shown in red). *n* = 5 mice per group. Scale bar, 200 µm (a). Data are mean ± s.e.m. (unpaired two-sided t-tests (a)).
