# Supplementary material for: Rectification of radiotherapy-induced cognitive impairments in aged mice by reconstituted Sca-1+ stem cells from young donors
Source: J Neuroinflammation. 2020 Feb 7;17:51. doi: 10.1186/s12974-019-1681-3 (PMC7006105; doi:10.1186/s12974-019-1681-3)
Supplement: Supplementary file 9 — Figure S9.Reconstituted human CD34+HSCs migrate from the BM to the brain. (a) Immunostaining and quantification of human Ku-80+/CD45+ cells in various different regions of the mouse brain. n = 5 mice. Scale bar, 200 μm (a). Data are mean ± s.e.m. (one-way ANOVA with Tukey’s multiple comparisons test tests (a)). (DOCX 121 kb) [file 12974_2019_1681_MOESM9_ESM.docx]

**
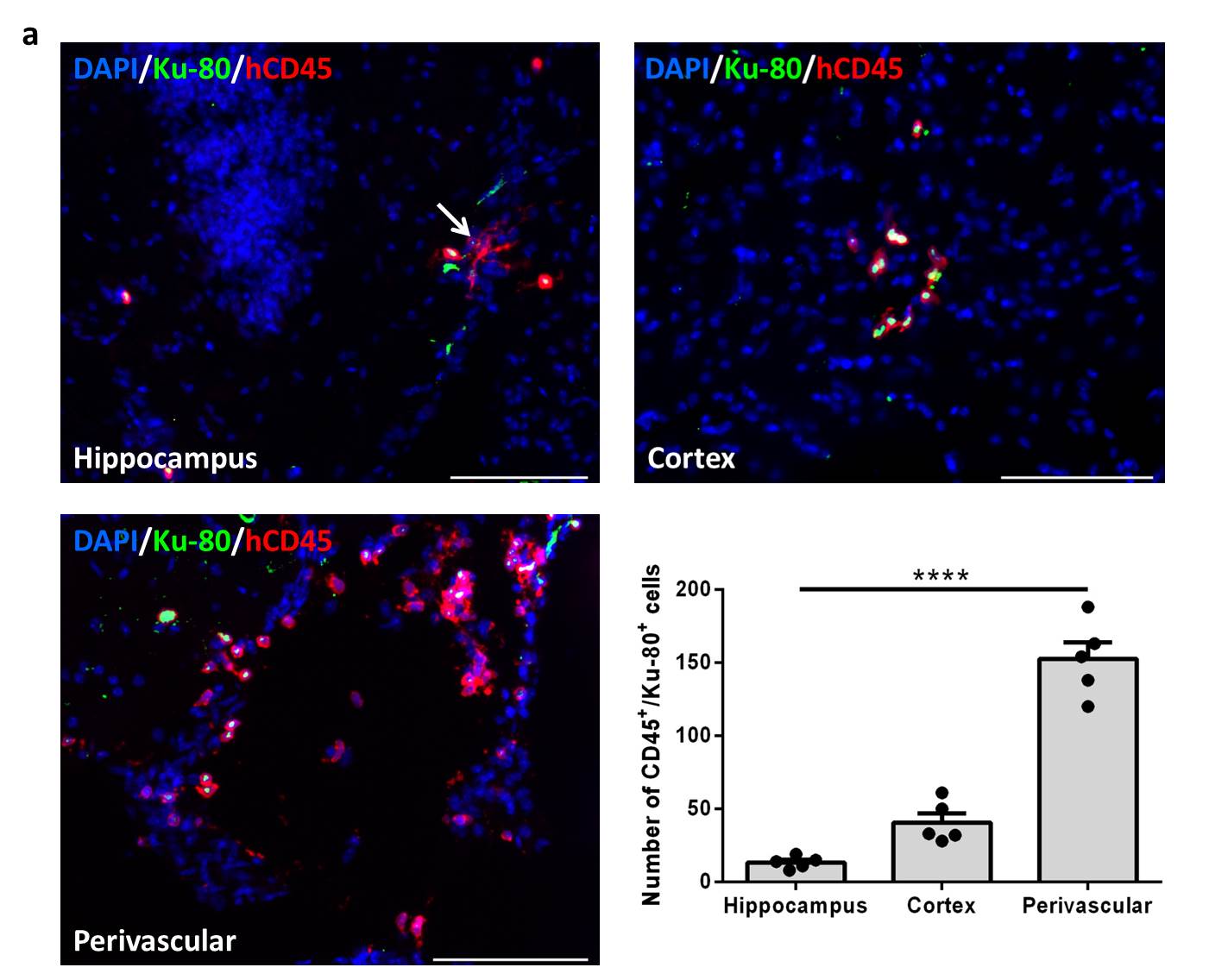
**

*Figure S9: Reconstituted human CD34^+^ HSCs migrate from the BM to the brain.* (a) Immunostaining and quantification of human Ku-80^+^/CD45^+^ cells in various different regions of the mouse brain. *n* = 5 mice. Scale bar, 200 µm (a). Data are mean ± s.e.m. (one-way ANOVA with Tukey's multiple comparisons test tests (a)).
